# *Utf1* contributes to intergenerational epigenetic inheritance of pluripotency

**DOI:** 10.1038/s41598-017-14426-5

**Published:** 2017-11-03

**Authors:** Qiuye Bao, Amir Morshedi, Fulu Wang, Sharma Bhargy, Konstantin Pervushin, Wei-Ping Yu, Peter Dröge

**Affiliations:** 1 School of Biological Sciences, Nanyang Technological University, 60 Nanyang Drive, Singapore, 637551 Singapore; 2 Animal Gene Editing Laboratory, Biological Resource Centre, Agency for Science, Technology and Research (A*STAR), Singapore, 138673 Singapore; 3 Nanyang Institute of Structural Biology, Nanyang Technological University, 59 Nanyang Drive, Singapore, 637551 Singapore; 4 Institute of Molecular and Cell Biology, Agency for Science, Technology and Research (A*STAR), Singapore, 138673 Singapore; 5 Present Address: Institute of Diabetes and Regeneration Research, Helmholtz Zentrum München, 85764 Neuherberg, Germany

## Abstract

Undifferentiated embryonic cell transcription factor 1 (Utf1) is expressed in pluripotent embryonic stem cells (ESCs) and primordial germ cells (PGCs). *Utf1* expression is directly controlled by pluripotency factors Oct4 and Sox2, which form a ternary complex with the *Utf1* enhancer. The Utf1 protein plays a role in chromatin organization and epigenetic control of bivalent gene expression in ESCs *in vitro*, where it promotes effective cell differentiation during exit from pluripotency. The function of *Utf1* in PGCs *in vivo*, however, is not known. Here, we report that proper development of *Utf1* null embryos almost entirely depends on the presence of functional *Utf1* alleles in the parental germline. This indicates that Utf1’s proposed epigenetic role in ESC pluripotency *in vitro* may be linked to intergenerational epigenetic inheritance *in vivo*. One component - or at least facilitator - of the relevant epigenetic mark appears to be Utf1 itself, since *Utf1*-driven tomato reporter and Utf1 are detected in mature germ cells. We also provide initial evidence for a reduced adult testis size in *Utf1* null mice. Our findings thus point at unexpected functional links between the core ESC pluripotency factor network and epigenetic inheritance of pluripotency.

## Introduction

Undifferentiated embryonic cell transcription factor 1 (Utf1) is primarily expressed in pluripotent embryonic stem cells (ESCs) and primordial germline cells (PGCs)^1–3^. General interest in Utf1 resulted from the demonstration that *Utf1* expression in mouse and human cells is directly regulated by core pluripotency factors Oct4, Sox2, and most likely Nanog, which form a ternary complex with the *Utf1* enhancer^4–6^. Furthermore, *Utf1* expression is a reliable early marker for and potent facilitator of *ex vivo* and *in vivo* cell reprogramming to full pluripotency^6–11^.

A number of molecular functions have been assigned to Utf1 in ESCs, such as transcription factor^1^, chromatin organizer^12^, and epigenetic factor controlling H3K27me3 deposition at bivalent genes via binding to thousands of loci around transcriptional start sites^13^. The latter is particularly interesting because it connects the pluripotency core to the polycomb-repressive complex 2 (PRC2) network, and the deposition of epigenetic chromatin marks in ESCs. These data, in conjunction with *Utf1* knock-down (kd) and knock-out (ko) mouse ESCs studies, pointed at an important regulatory role for Utf1 during exit from ESC pluripotency en route to effective cell differentiation^12,13^.

A subsequent *Utf1* ko (*Utf1*^(−/−)^) mouse study, however, revealed that Utf1 is not critical for pluripotency, because viable and fertile *Utf1*^(−/−)^ mice can be obtained^14^. A noticeable *Utf1*^(−/−)^ phenotype reported in this study was developmental delay during embryogenesis that often caused smaller pups and neonatal death, which was attributed to reduced placental growth. However, the delay can be resolved during neonatal growth, thus resulting in phenotypically normal adult *Utf1*^(−/−)^ mice^14^. The *in vivo* relevance of Utf1-controlled maintenance of pluripotency, as proposed for ESCs *in vitro*, therefore remains an unresolved issue^13,15^.

Here, we report important insights into this topic by employing novel *Utf1-tomato* pluripotency reporter mice that carry either *Utf1*^(−/+)^ or *Utf1*^(−/−)^ ko alleles due to the *tdTomato* replacement of *Utf1* exon 1. Strikingly, by genotyping of embryos at 16 days post coitum (dpc), we provide evidence that a regulatory role of *Utf1* in pluripotency maintenance is linked to an intergenerational epigenetic mark in parental germ cells. Based on our data and in conjunction with information available in the literature, we propose a model in which the Utf1 protein and/or *Utf1* mRNA is a key component or a facilitator of such an epigenetic mark. We also made an interesting initial finding that hinted at a negative correlation between adult testis vascularization and size with the *Utf1*^(−/−)^ genotype.

## Results

### *Utf1-tomato* reporter mice strain for pluripotency

In order to probe into the *in vivo* relevance of the proposed Utf1-controlled maintenance of pluripotency^13,15^, we generated *Utf1-tomato* pluripotency reporter mice in which the *tdTomato* gene replaced a large segment of *Utf1* exon 1 on chromosome 7. The inserted *tdTomato* gene carries two translational stop codons (Figure S1). Furthermore, the knock-in (ki) generated a +2 frameshift with respect to the unaltered part of the *Utf1* coding sequence. Hence, the ki resulted in a *Utf1* null allele, and we refer to the corresponding hetero- or homozygous transgenic animals as either *Utf1*^(−/+)^*-tomato* or *Utf1*^(−/−)^*-tomato* mice, respectively, and to the non-transgenic wild-type animals as *Utf1*^(+/+)^.

**Figure 1:**
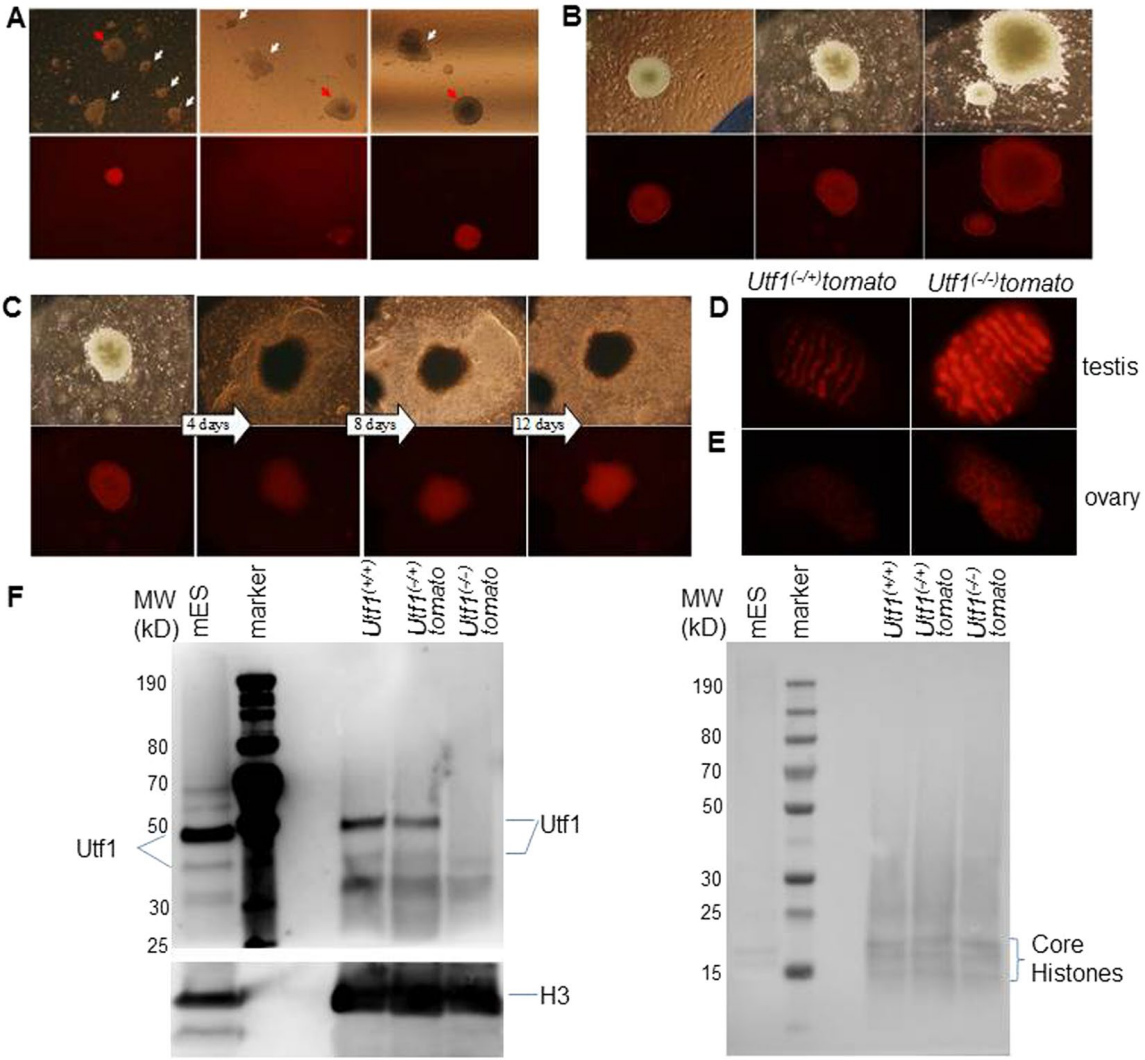
Characterization of *Utf1-tomato* pluripotency reporter mice. **(A)** iPSC colonies with ESC-like morphologies generated from *Utf1-tomato* mice begin to express the reporter around 14 days after the first “Yamanaka” factors transduction. (**B)** Expression of the tomato reporter in established iPSC colonies. Around 21 days after the “four factors” transduction, iPSC colonies show sustained and strong tdTomato expression. (**C)** An individual iPSC colony was followed during 12 days of DMSO-induced differentiation. (**D)** *Utf1-tomato* expression in mouse embryonic testis around 15 dpc, left: *Utf1*^(−/+)^*-tomato* embryonic testis; right: *Utf1*^(−/−)^*-tomato* embryonic testis. (**E)** Expression of *Utf1-tomato* in *Utf1*^(−/+)^*-tomato* (left) and *Utf1*^(−/−)^*-tomato* (right) ovaries at 15 dpc. (**F)** Utf1 protein expression in embryonic testes determined by Western Blotting (WB). A total protein lysate from mouse ESCs was used as control (left lane). The left panel shows that Utf1 is detectable in *Utf1*^(+/+)^ and *Utf1*^(−/+)^*-tomato* embryonic testis, but not in lysates from *Utf1*^(−/−)^*-tomato* testis extracted from the same pregnant female that carried the *Utf1*^(−/+)^*-tomato* embryo. Bottom panel: WB showing equal amounts of histone H3 as loading control. Right panel: Ponceau S-staining of PVDF membrane as second loading control. The signals from core histones are indicated on the right.

First, we tested the functionality of the pluripotency reporter *in vitro* and derived fibroblasts from tail-tips of *Utf1*^(−/+)^*-tomato* mice. We subjected them to our established reprogramming protocol^10,16^ and found that clear fluorescent signals appeared in induced pluripotent stem cell (iPSC) colonies two weeks after “Yamanaka” factors transduction. The intensity of tdTomato expression correlated with iPSC/ESC colony morphology; i.e. colonies not resembling pluripotent stem cells showed weak or no detectable reporter expression (Fig. 1A, white arrows**)**. Seven days later, iPSC colonies with sustained and strong reporter signals became visible (Fig. 1B).

We further tested our *Utf1* reporter by DMSO-induced iPSC differentiation. While Tomato expression was maintained in the centre of iPSC colonies for several days, the outgrowing differentiated cells with fibroblast-like morphology had completely lost reporter signals (Fig. 1C). This had been observed earlier with different *Utf1* reporter constructs^2,6^. Interestingly, the size of the fluorescent section in the centre of each colony did not change during 12 days of induced differentiation, indicating that cell proliferation and DNA replication at the edges of the central section triggers iPSC differentiation and concomitant down-regulation of *Utf1*. These results are in line with recent evidence for a functional link between the cell cycle and control of cell fate decisions in pluripotent stem cells^17^.

It has previously been shown that Utf1 is strongly expressed in gonocytes of rat testes during the embryonic and neonatal stage^18^. Furthermore, the highest expression level of *Utf1* was observed in epiblast stage embryos (E6.5) and in PGCs (E12.5) in mice^2^. In order to verify that our reporter faithfully recapitulated known *in vivo Utf1* expression patterns, we isolated testes from embryos at 15.5 dpc and found tdTomato expression in the cords of *Utf1*^(−/+)^*-tomato* and, at the expected higher levels, in *Utf1*^(−/−)^*-tomato* embryonic testes (Fig. 1D). In agreement with an earlier report^2^, we also detected reporter expression in PGCs of ovaries isolated at the same embryonic stage, although the signal intensities were clearly weaker (Fig. 1E).

We next determined Utf1 expression in embryonic testes by Western blotting and included mouse ESCs as positive control. It should be noted that the 36 kDa Utf1 protein is known to exhibit unusual mobility patterns in Western blots due to post-translational modification, such as phosphorylation^19^. We found that the vast majority of the protein was modified in embryonic *Utf1*^(+/+)^ and in *Utf1*^(−/+)^*-tomato* testes, and that the protein was absent in all lysates prepared from testes obtained from different *Utf1*^(−/−)^*-tomato* embryos (Fig. 1F; Figure S2). The protein was also not detectable in embryonic mouse kidneys, irrespective of the genotype (Figure S2A). The latter result is in line with the known highly tissue-restricted *Utf1* expression pattern during embryogenesis^2,14^. Taken together, we conclude that our *Utf1-tomato* reporter is a reliable indicator of *Utf1* expression, and, as predicted, *Utf1*^(−/−)^*-tomato* mice do not express the Utf1 protein.

### A functional parental *Utf1* allele is important for *Utf1*^(−/−)^ embryonic development

*Utf1* is expressed in mouse and human PGCs, during oogenesis, and in the spermatogonial stem cell niche in testes^3,18,20,21^. The latter maintains the ability to self-renew and differentiate into functional spermatozoa^22^. However, functions for Utf1 in these important cell types are not known. Based on this well-established *Utf1* expression pattern and the proposed role of *Utf1* in epigenetic regulation of bivalent genes and maintenance of pluripotency in ESCs *in vitro*^13,15^, we became interested in interrogating whether *Utf1* genotype frequencies of progenies might be affected by the parental *Utf1* genotype. In order to prevent confounding effects due to neonatal developmental delays and associated postnatal lethality that we and others observed with *Utf1*^(−/−)^*-tomato* pups^14^, we decided to genotype progenies at embryonic stage 16 dpc.

**Figure 2:**
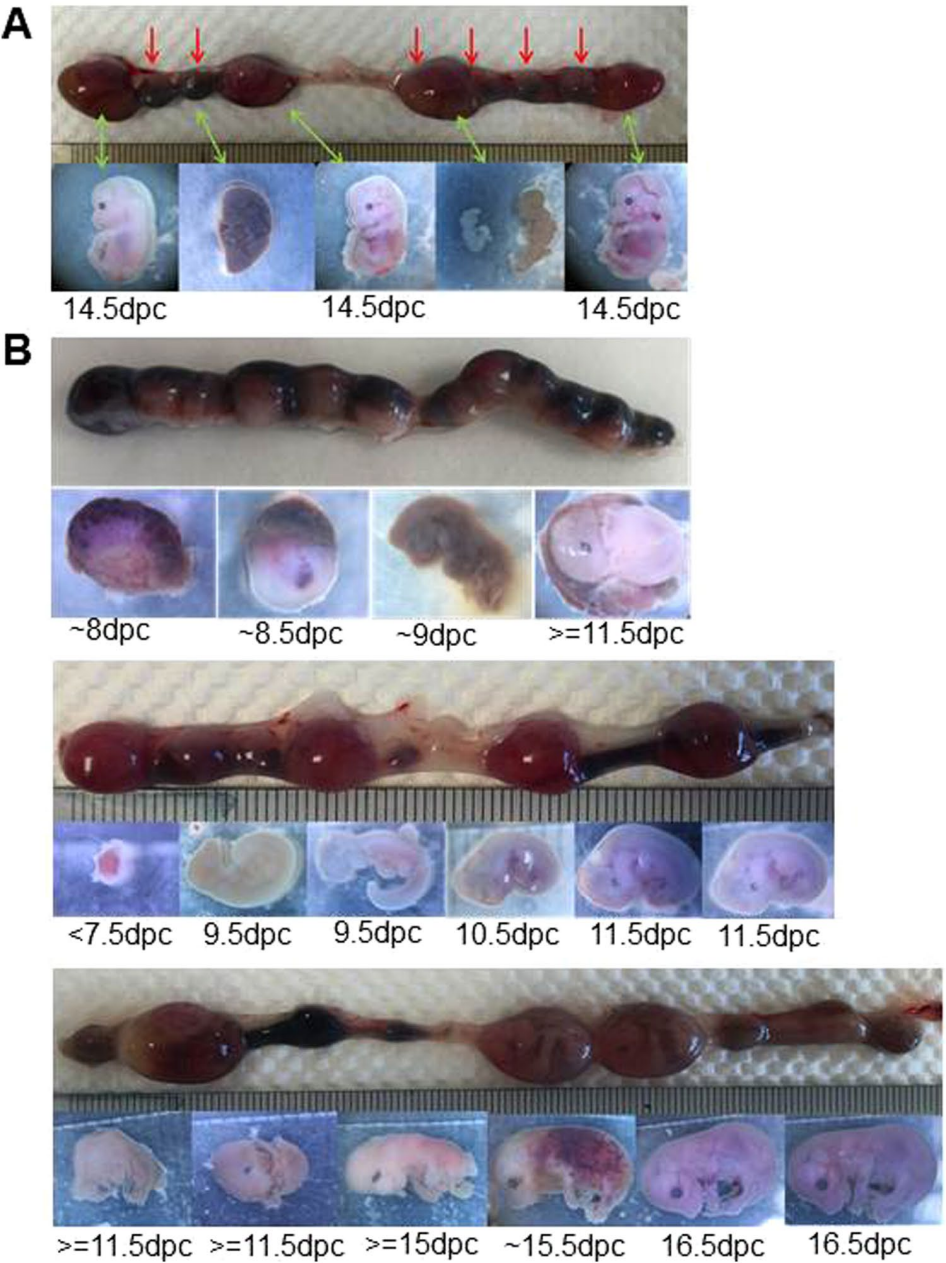
Early embryonic developmental arrest. (**A**) Embryos arrested at different developmental stages from breeding *Utf1*^(−/+)^*-tomato* females and *Utf1*^(−/−)^*-tomato* males at 16 dpc. Embryos marked by red arrows in the uterus could not be isolated (upper panel). **(B)** Embryos from breeding *Utf1*^(-/-)^-*tomato* females and *Utf1*^(−/−)^*-tomato* males were developmentally arrested at different stages, which can be seen in the uterus (at 20dpc) (upper panels) and with isolated embryos (lower panels).

Breeding *Utf1*^(−/+)^-*tomato* males with *Utf1*^(−/+)^-*tomato* females resulted in a 1:2:1 Mendelian *Utf1* ratio with an average of 8.23 embryos/cage, which is in the normal litter size range for wild-type C57BL/6 J mice (Table 1). This result is in agreement with *Utf1*^(−/+)^ breeding data from a previous study^14^ and indicated that *Utf1*^(−)^ sperm cells produced by *Utf1*^(−/+)^*-tomato* males are functionally equivalent to *Utf1*^(+)^ sperms, i.e. they produced the expected number of *Utf1*^(−/+)^-*tomato* and *Utf1*^(−/−)^-*tomato* embryos. However, breeding *Utf1*^(−/−)^*-tomato* males with *Utf1*^(−/+)^*-tomato* females produced embryos in a *Utf1*^(−/+)^-*tomato*:*Utf1*^(−/−)^*-tomato* ratio of about 2:1, instead of the expected 1:1 Mendelian ratio. We found that the reduced number of *Utf1*^(−/−)^*-tomato* embryos was statistically highly significant (p = 0.0012) (Table 1). As expected, the average number of embryos/cage was also reduced to 7.6. This reduction was not caused by a decrease in the reproductive potential of *Utf1*^(−/−)^*-tomato* reporter males, since, on average, they produced 4.9 *Utf1*^(−/+)^*-tomato* embryos/cage, which is even higher than the average 4.4 *Utf1*^(−/+)^*-tomato* embryos/cage produced by *Utf1*^(−/+)^*-tomato* males (Table 1). Instead, we frequently observed embryos which were arrested at various developmental stages (Fig. 2A). Hence, developmental arrest, probably in combination with uterine resorption, accounts for the observed reduction in the expected number *Utf1*^(−/−)^*-tomato* embryos.

**Table 1:** Breeding outcomes for *Utf1* genotypes in 16 dpc embryos.

**Cage No**	***Utf1*** ^(−/+)^ **(F) X** ***Utf1*** ^(−/+)^ **(M)**			***Utf1*** ^(−/+)^ **(F) X** ***Utf1*** ^(−/−)^ **(M)**	
	***Utf1*** ^(+/+)^	***Utf1*** ^(−/+)^	***Utf1*** ^(−/−)^	***Utf1*** ^(−/+)^	***Utf1*** ^(−/−)^
**1**	2	3	4	6	4
**2**	0	6	4	3	2
**3**	0	2	3	0	2
**4**	0	2	3	9	2
**5**	3	2	3	3	1
**6**	2	4	3	5	4
**7**	4	4	0	3	5
**8**	2	5	2	3	2
**9**	3	3	1	7	3
**10**	4	3	0	5	0
**11**	4	5	2	7	4
**12**	1	6	2	7	2
**13**	1	3	5	7	2
**14**	2	4	2	2	4
**15**	2	5	0	5	4
**16**	2	8	0	5	2
**17**	2	4	3	3	2
**18**	1	4	2	7	2
**19**	2	4	1	8	1
**20**	1	11	0	3	6
**21**	1	5	3		
**22**	3	6	1		
**23**	4	2	3		
**24**	0	3	1		
**25**	1	4	2		
**26**	1	7	1		
**total**	***48***	***115***	***51***	***98***	***54***
**Average genotype No/cage**	***1***.***85***	***4***.***42***	***1***.***96***	***4***.***90***	***2***.***70***

The predicted 1:1 occurrence of the two expected genotypes that resulted from *Utf1*^(−/+)^-*tomato* (Female) X *Utf1*^(−/−)^-*tomato* (Male) breeding was statistically analysed by the unpaired t-test with Welch’s correction. The mean values (n = 20) for both genotypes showed a highly significant difference (p value = 0.0012).

Together these data indicated that the 16 dpc embryonic development of *Utf1*^(−/−)^*-tomato* mice critically depended on the presence of at least one functional *Utf1* allele in the paternal germ line. In order to test whether this also applied to the maternal germ line, we next examined progenies from breeding *Utf1*^(−/−)^*-tomato* males with *Utf1*^(−/−)^*-tomato* females. We found that out of fifty six breeding cages, only eight produced litters and a total of 14 viable pups, i.e. on average only 1.75 pups per litter. Examination of several pregnant females and their *Utf1*^(−/−)^*-tomato* embryos from this breeding revealed that they were developmentally arrested between 8 to 16 dpc, or earlier (Fig. 2B). This arrest was not caused by an uterine phenotype associated with *Utf1*^(−/−)^*-tomato* females, because breeding them with *Utf1*^(−/+)^*-tomato* males produced, on average, 7.1 viable pups/cage, which is in the range of the 6 to 7.6 pups/cage obtained with heterozygous *Utf1*^(−/+)^*-tomato* females **(**Table S1).

### *Utf1* is expressed during parental gametogenesis

**Figure 3:**
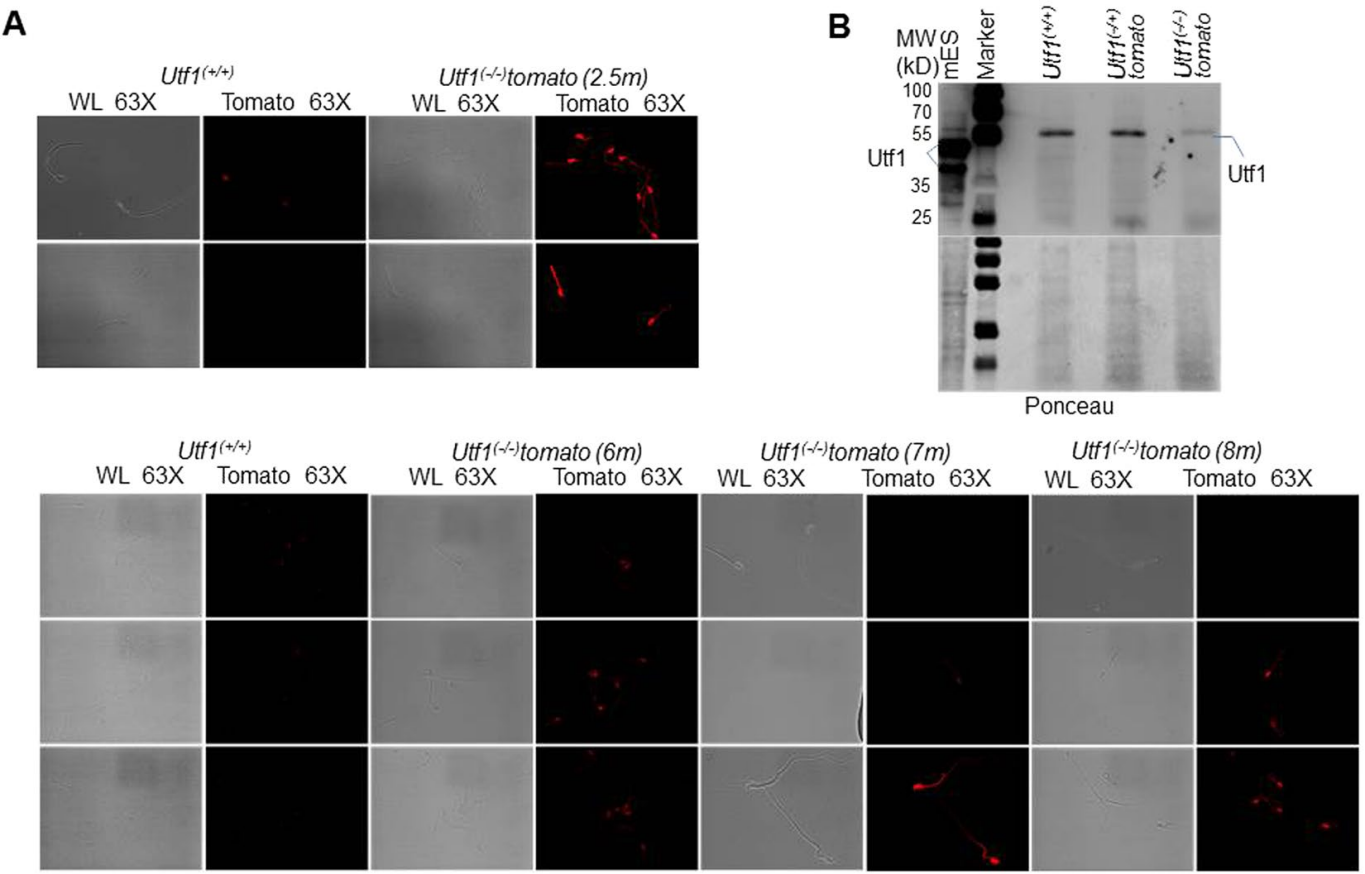
Tomato and Utf1 protein expression in sperm cells. **(A)** Expression of Tomato protein in sperm cells extracted from *Utf1*^(−/−)^*-tomato* males of different ages. Compared with control (sperm cells from age-matched *Utf1*^*+/+*^ mice), sperms from 2.5 months old mice showed strong tdTomato signals in the head and mid-piece regions. The bottom panels show that signal intensity became much more varied in 6 to 8 months old mice (see also Figure S3A). Magnification: 63X. (**B)** Utf1 protein can be detected in sperm cells of *Utf1*^(+/+)^ and *Utf1*^(−/+)^*-tomato* mice. A lysate from mouse ESCs was used as positive control. The upper panel shows the WB result, and the lower panel depicts the corresponding membrane after Ponceau S-staining as loading control. Note that a faint non-specific band migrates above the specific Utf1 signal (see also Figure S3B).

Together our data showed that the full development potential of *Utf1*^(−/−)^*-tomato* embryos at 16 dpc critically depended on the parental *Utf1* genotype, i.e. the presence of at least one functional maternal and paternal *Utf1* allele. This can be explained by a role of *Utf1* in intergenerational epigenetic inheritance^23,24^. Since it is known that Utf1 is expressed in the spermatogonial stem cell niche^21,25^, we hypothesized that a component or a facilitator of relevant intergenerational epigenetic mark(s) deposited in paternal germ cells is the Utf1 protein and/or mRNA. We therefore tested whether *Utf1* promoter activity leads to the presence of fluorescent reporter in mature *Utf1*^(−/−)^*-tomato* sperm cells. Compared to an almost undetectable background in sperm cells isolated from *Utf1*^(+/+)^ males, very strong tdTomato signals were detectable in the head and mid-piece regions of all examined sperm cells derived from two different *Utf1*^(−/−)^*-tomato* males at the age of 2.5 months (Fig. 3A; Figure S3A). We also analysed whether the reporter expression is maintained in older *Utf1*^(−/−)^*-tomato* males. Interestingly, we found that signal intensities were reduced and nearly lost in a number of sperm cells from males at the age of six to eight months (Fig. 3A; Figure S3A).

In order to detect the Utf1 protein in sperm cells, we performed Western blotting and detected the Utf1 protein migrating slightly below a non-specific background signal in sperm lysates prepared from two different pairs of *Utf1*^(+/+)^ and *Utf1*^*(+/-)*^ males, but not in lysates from *Utf1*^(−/−)^*-tomato* males (Fig. 3B; Figure S3B). Interestingly, Utf1 has recently also been detected in various age groups of mouse metaphase II oocytes by both transcriptome and proteome analyses^20^. Combined these data reveal that Utf1 protein and mRNA are present in maternal and paternal wild-type *Utf1* mouse germ cells.

### *Utf1* expression in PGCs and a potential role in testis development

**Figure 4:**
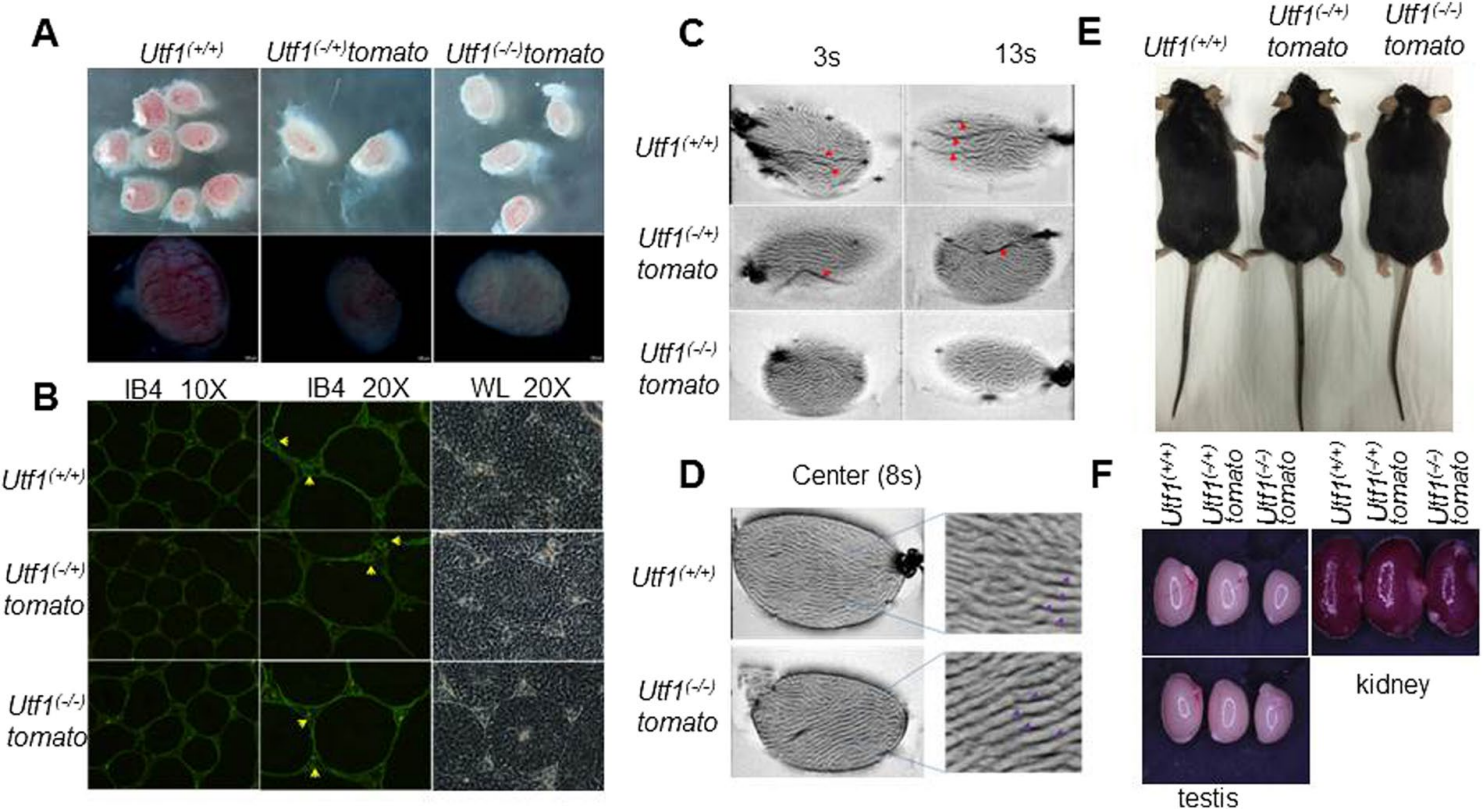
Testicular vasculature and size in *Utf1*^(−/−)^*-tomato* testis. **(A)** Light microscopic images of embryonic testes. Compared with *Utf1*^(+/+)^ and *Utf1*^(−/+)^*-tomato* testes, *Utf1*^(−/−)^*-tomato* testes exhibit fewer fully developed capillaries on the surface. (**B)** Immunofluorescence staining of mouse adult testis sections with IB4 (green) revealed that capillaries (yellow arrows) of *Utf1*^(+/+)^ and *Utf1*^(−/+)^*-tomato* testes are better developed than those in *Utf1*^(−/−)^*-tomato* testes. (**C)** and **(D)** MRI analysis of adult testes of different genotypes obtained from the same males analysed in (B). Gradient echo images (FLASH, TR/TE = 1900/14 ms, resolution = 27 × 55 µm^2^, slice thickness = 0.4 mm, NA = 5, flip angles = 30°). 3 s and 13 s vertical sections of 2D MRI movies showed *Utf1*^(+/+)^ and *Utf1*^(−/+)^*-tomato* testes have more pronounced and thicker blood vessels (red arrows) than *Utf1*^(−/−)^*-tomato* testes. The central images revealed that *Utf1*^(+/+)^ and *Utf1*^(−/−)^*-tomato* testes have similar seminiferous tubules morphologies (purple arrows in (D). (See also SI movies for full organ examination). **(E)** *Utf1*^(+/+)^, *Utf1*^(−/+)^*-tomato* and *Utf1*^(−/−)^*-tomato* pluripotency reporter mice from the same litter at 6 months of age are indistinguishable in appearance and size. (**F)** Testes from the adult *Utf1*^(−/−)^*-tomato* mice shown in (**E**) are much smaller than those from *Utf1*^(+/+)^ and *Utf1*^(−/+)^*-tomato* mice, while the kidneys exhibited no size difference (see also Figure S6).

Based on the high *Utf1-tomato* reporter activity observed in PGCs of *Utf1*^*(+/−)*^ and *Utf1*^(−/−)^*-tomato* testes (Fig. 1D), we decided to examine this organ more carefully. *Utf1*^(−/+)^-*tomato* reporter mice were bred, and a pregnant female sacrificed at 15.5 dpc for the examination of testes from four *Utf1*^(+/+)^ embryos. As expected, we found fully developed surface blood vessels on all eight organs (Fig. 4A). However, the four embryonic testes from *Utf1*^(−/−)^*-tomato* embryos that could also be obtained from the same pregnant female showed a marked reduction in visible blood vessels, which appeared much thinner and resulted in rather pale organs. This phenotype was also present, albeit less pronounced, with the two *Utf1*^(−/+)^*-tomato* embryonic testes that we could examine from the same pregnant female (Fig. 4A). Such genotype-specific differences in vasculature were not observed with embryonic ovaries from embryos that we extracted from another pregnant *Utf1*^(−/+)^-tomato female (Figure S4A).

To probe further into the possibility of compromised testicular vasculature development in the *Utf1*^(−/−)^*-tomato* embryos, we examined the hemoglobin α contents in embryonic testes. Using SDS-PAGE combined with mass spectrometry, we found that the protein was present in high amounts in *Utf1*^(+/+)^ *and Utf1*^(−/+)^*-tomato* testes, but clearly reached the detection limit of this method in *Utf1*^(−/−)^*-tomato* organs. These genotype-specific differences in blood content were not obvious in other embryonic organs, such as kidney, heart or ovaries (Figure S4B and S4C; Figure S5).

We next examined whether differences in vasculature also manifested in adult organs. Histological sections of adult testes obtained from three litter mates were analyzed by immunofluorescence using Isolectin IB4 Alexa Fluor 488, which is a marker for seminiferous tubules basement membranes and capillaries in the interstitial tissue^26^. Compared to the large and clearly visible capillaries in testes of *Utf1*^(+/+)^ mice, those in *Utf1*^(−/−)^*-tomato* organs were much thinner and their appearance less frequent (Fig. 4B).

In order to confirm these results by a different method, we employed magnetic resonance imaging (MRI) to examine these testes. It confirmed that major capillaries of *Utf1*^(−/−)^*-tomato* adult testis are much thinner and less pronounced than those found in *Utf1*^(−/+)^*-tomato* and *Utf1*^(+/+)^ organs (Fig. 4C; SI Movies 1–3). Based on our MRI analysis, we did not detect obvious genotype-specific differences in seminiferous tubules morphology (Fig. 4D; SI Movies 1–3**)**. It appears, therefore, that lack of *Utf1* expression in PGCs could affect testicular vasculature development during embryogenesis, which may also influence adult organ vasculature.

An impaired organ vascularization during development is known to have the potential to affect adult organ size^27^. We therefore examined the size of testes of adult *Utf1*^(−/−)^*-tomato* reporter mice. While some of these mice showed an overall reduced body size at birth, as reported earlier in a different genetic background^14^, their appearance became indistinguishable from wild-type and heterozygous littermates during postnatal development (Fig. 4E, Figure S6A,B). We found that the size and weight of testes was substantially reduced compared with testes of their *Utf1*^(+/+)^ and *Utf1*^(−/+)^*-tomato* littermates (Fig. 4F, Figure S6A–D). We observed this with all ten testes from five *Utf1*^(−/−)^*-tomato* males that we were able to obtain together with the corresponding male littermate genotypes. We also examined kidneys and found no size or shape differences (Fig. 4F, Figure S6A,B). Hence, the correlation between reduced testis size and *Utf1*^(−/−)^*-tomato* genotype appears to be organ-specific.

We next examined the size of *Utf1*^(−/−)^*-tomato* embryonic testes and found differences in conjunction with the already described vascularization impairment at 18.5 dpc (Figure S7). However, since *Utf1*^(−/−)^*-tomato* embryos occasionally appear to be smaller than *Utf1*^(−/+)^*-tomato* and *Utf1*^(+/+)^ embryos^14^, we cannot exclude that a general growth delay contributed to smaller testes at this stage. Interestingly, however, we observed that *Utf1*^(−/−)^*-tomato* embryos frequently exhibited a very pale appearance, indicating that organ vasculature development problems in the *Utf1* null background could reach beyond testes (Figure S8). Together our data provide initial evidence for a potential role of *Utf1* expression in PGSs in connection with testis development.

## Discussion

The main result of our study on the *in vivo* function of *Utf1* is that depending on the presence or absence of functional parental *Utf1* alleles, *Utf1*^(−/−)^*-tomato* embryos in the next generation either develop normally or arrest at various stages of development, respectively. Such phenomenon can best be explained by intergenerational epigenetic inheritance rather than a classical paramutation^23,24,28^. In the latter case, we expect that the embryonic development phenotype should become detectable in F1 homozygous wild-type *Utf1*^(+/+)^ embryos generated by *Utf1*^(−/+)^*-tomato* mice^29^. This is clearly not the case (Table 1). Furthermore, we demonstrated that *Utf1* promoter-driven Tomato and the Utf1 protein is present in sperm cells of *Utf1*^(−/−)^*-tomato* and *Utf1* wild-type males, respectively, and Schwarzer *et al.*^20^ identified the Utf1 protein and mRNA in stage II mouse oocytes.

**Figure 5:**
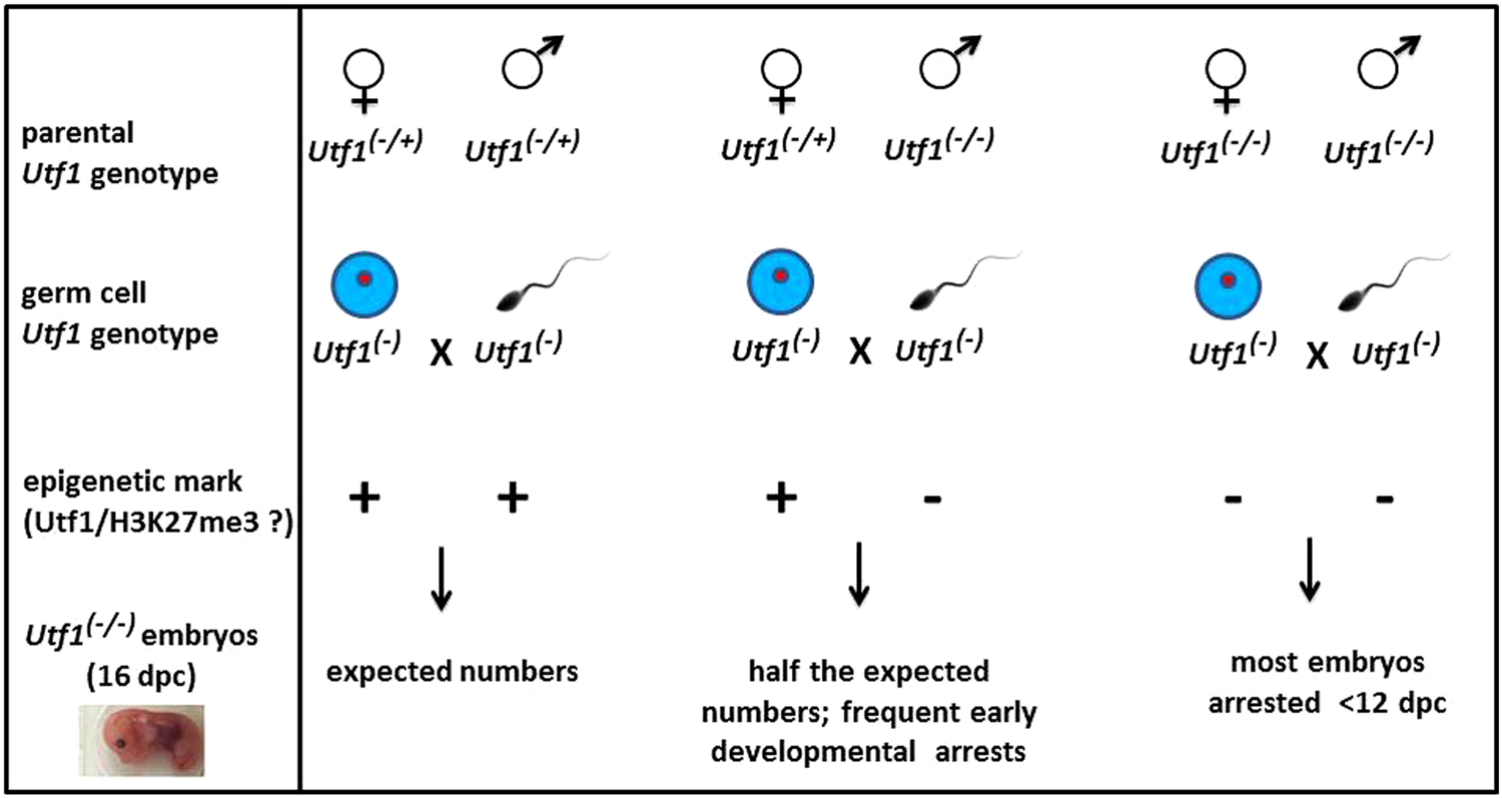
Model of an epigenetic role for *Utf1* in intergenerational inheritance of pluripotency. *Utf1*^(−)^*-tomato* sperm cells produced by *Utf1*^(−/−)^*-tomato* males lack a relevant epigenetic mark that is present in *Utf1*^(−)^*-tomato* sperm cells, which are produced in *Utf1*^(−/+)^*-tomato* testes. Such a mark also plays a role in the female germ line, because the development of the vast majority of embryos became arrested when *Utf1*^(−/−)^*-tomato* males were bred with homozygous *Utf1*^(−/−)^*-tomato* females, instead of *Utf1*^(−/+)^-*tomato* females.

Based on our data and information in the literature, we propose a model in which *Utf1*^(−)^*-tomato* sperm cells produced by *Utf1*^(−/−)^*-tomato* males lack a relevant epigenetic mark that is present in *Utf1*^(−)^*-tomato* sperms, which are produced in *Utf1*^(−/+)^*-tomato* testes (Fig. 5). It is clear from our data that such a mark also plays a role in the female germ line, because the development of the vast majority of embryos became arrested when *Utf1*^(−/−)^*-tomato* males were bred with homozygous *Utf1*^(−/−)^*-tomato* females, instead of *Utf1*^(−/+)^-*tomato* females (Fig. 5). Some of the earliest arrests may occur when the pluripotent primitive ectoderm begins to convert into the three primary germ layers^30^. Since the tomato reporter as well as the endogenous Utf1 protein is detectable in both mature sperm cells (this study) and oocytes^20^, we propose that a critical component of this epigenetic mark could be the Utf1 protein itself, most likely bound to parental DNA via its Myb domain^2,13,19,31^. The fact that the core pluripotency factors Oct4, Sox2, and Nanog, which control *Utf1* expression in ESCs^2,5^, are also expressed in PGCs^23^ implies that the pluripotency core is directly connected to the proposed intergenerational epigenetic inheritance mediated by *Utf1*.

Jia *et al*.^13^ recently reported that Utf1 regulates PRC2 loading and H3K27me3 levels in mouse ESCs, thereby contributing to the control of bivalent gene expression. Furthermore, the study provided evidence that Utf1 is involved in the control of the stability of messenger RNAs transcribed from incompletely silenced bivalent genes in ESCs. Functionally, this dual gene regulatory role of Utf1 in mouse ESCs appears to be critical for the induction of proper ESC differentiation pathways *in vitro*^13^. Interestingly, recent studies indicated that H3K27me3 epigenetic marks are also present at bivalent domains of developmental regulatory genes in mouse PGCs, which are highly similar to the domains marked in ESCs^32,33^. In *Drosophila melanogaster*, H3K27-trimethylated nucleosomes remain heritably associated with silenced *hox* genes and can carry this epigenetic memory through multiple rounds of DNA replication^34,35^. Furthermore, this type of histone modification is also found at important gene regulatory regions in human sperm cells^36–38^. Since Utf1 is present in PGCs, spermatogonial stem cells, and functional sperm cells (Fig. 3A,B), we think it is possible that the protein contributes there to H3K27me3 deposition in a manner similar to its role in ESCs^13^. The same reasoning applies to oocytes, where Utf1 is also present^20^. It is also worth noting that the *Utf1* gene itself is controlled by H3K27me3 epigenetic modification^8^, thus potentially indicating an epigenetic regulatory feedback mechanism.

Other, mutually not exclusive possibilities are that the parental Utf1 protein delivered by functional germ cells engages in interactions with target genes, components of the mRNA-decapping complex^13^ or, perhaps, other RNA modifying proteins, and thereby contributes to transcriptional or post-transcriptional regulation of gene expression during, for example, zygote-to-embryo transition^39^. In either case, such a contribution would ultimately become critical in the context of exit from pluripotency and efficient initiation of cell differentiation programs in the next generation embryos. Finally, it is also possible that only the *Utf1* mRNA present in germ cells might play a functional role as an epigenetic mark.

Our study also presented initial findings which hinted at a contribution of Utf1 to the development of testicular vasculature and adult testes size. Clearly, this needs to be confirmed in other *Utf1* ko genetic backgrounds. Given the high expression levels of *Utf1* specifically in PGCs in male embryos, we think an interesting question to answer in the future is whether Utf1 is directly involved in cell signaling during testicular development.

In summary, because *Utf1* is directly regulated by Oct4, Sox2, and, most likely, Nanog^6^, our data established the first example for intergenerational epigenetic inheritance of pluripotency mediated, at least in part, by a factor belonging to the core ESC pluripotency network. It will be very interesting to dissect in the future the molecular function(s) of the proposed Utf1-mediated epigenetic mark(s).

## Materials and Methods

### Generation of *Utf1-tomato* reporter mice

We generated an *Utf1* gene target vector that carries a short arm of *Utf1* homology, a tandem dimeric *Tomato* (*tdTomato*) cassette (Clonetech) with two stop codons, an excisable selection cassette, a long arm of homology, and a thymidine kinase screening cassette (Figure S1A). Homologous recombination in ESCs inserted *Tomato* into *Utf1* exon 1, replacing the coding sequence for the first 173 amino acids which harbor its conserved DNA binding domain (Fukushima *et al*.^19^; Kooistra *et al*.^12^). The *Tomato* reporter is translated from the *Utf1* start codon. Hence, with two stop codons at the end of the *Tomato* cassette, expression of *Utf1* from a successfully targeted allele is not possible. The target vector was introduced into 129 ES cells and screened by PCR and Southern blotting for successful integration (data not shown). The genomic selection marker was subsequently excised, verified by PCR, and modified ES cells were injected into blastocysts. Resulting chimera were crossed with C57BL/6 J mice to generate germ-line-competent heterozygous *Utf1-tomato* mice. Heterozygous and homozygous animals were genotyped by PCR (Figure S1B), and sequences between arms of homology were verified by PCR/sequencing (Figure S1C; data not shown).

### Genotyping

*Utf1-tomato* animals were genotyped by PCR, using tail-tip DNA as template, using primers utf-F: 5′ TGTCCCGGTGACTACGTCTGATGCC 3′; utf-R: 5′ ATCGTCCCCCAATAGCCCCATGAG 3′; tmt-F: 5′ TCAGGGCTCCGCCCCTCC CCAGGAG 3′; tmt-R: 5′ ACTTGGATCCAAGCTGGACATCACCTCCCACAACG 3′. The PCR was run with following settings: 95 °C 2 min, 35 cycles (95 °C 30 s, 55 °C 30 s, 72 °C 1 min), 72 °C 5 min. To verify the nucleotide sequence between the arms of homology, we used primers UTF1f: 5′ TCTCTGGTGAGGCCACGCCTTG 3′; UTF1r: 5′ TCGGGGTA GACTGGG AGTCG 3′, and amplified with the following setting: 95 °C 2 min, 35 cycles (95 °C 30 s, 56 °C 30 s, 72 °C 2 min), 72 °C 5 min.

### IPSC reprogramming

Tail tips from *Utf1*^(−/+)^*-tomato* mice were used to generate fibroblasts *in vitro* for further induction to iPSCs. Viral supernatant from PLAT-E (Ectropic packaging cell line) cells, transfected with pMYc-based retroviral vectors^16^, was passed through 0.45 µm pore sized filters and supplemented with 4 μg/ml polybrene (Millipore). 6-well plates coated with 10 µg/ml fibronectin (r-fibronectin CH-296 from TAKARA) were centrifuged with the viral supernatant (no cells) for 60 min at 4000 rpm (4^o^C). 1 × 10^5^ cells were added to each well containing the viral supernatant. After transduction for two consecutive days, cells were cultured in general MEF medium until day 5. Cells were then detached and seeded into a 6-well plate coated with approximately 2 × 10^4^ inactivated MEF cells for each well. On day 7, the medium was replaced with mouse ESC medium (containing LIF). Culture medium was changed every day until iPSC colonies appeared. In order to differentiate generated iPSCs, 1% DMSO was added daily to differentiation medium (DMEM containing 20% FBS, without LIF) for the period of 12 days.

### Animal experiments

The culture and experiments using C57BL/6 J reporter mice were performed according to protocols approved by the Biological Resource Centre (IACUC: #161125). For breeding, 2 to 8 months old transgenic mice were mated as single pairs or initially in trios (1 male and 2 females) and, subsequently, pregnant females were moved to a new cage before females dropped their litter. For isolation of embryonic testes and embryos, pregnant females were euthanized by CO_2_ and embryos were genotyped by PCR. To analyze adult mouse testis, male mice (2–6 months) were sacrificed.

### Western Blotting

Proteins were extracted from embryonic testes or kidneys using a lysis buffer containing 10 mM Tris-HCl, pH7.4, 150 mM NaCl, 1 mM EDTA, 1% SDS in 1X PBS and 0.5 mM PMSF. The extracted samples were loaded on BoltTM 4–12% Bis-Tris Plus gradient gel (Invitrogen) and electrophoretically transferred to PVDF membrane. The protein loading was analyzed by Panceau S staining. After blocking with Super Block T20 (TBS) Blocking buffer (Thermo), the membrane was probed with primary antibody anti-Utf1 from rabbit (1:500 dilution, a24273 ABCAM). The antibody was detected with anti-rabbit HRP-conjugated secondary antibody (Dako). WB for mouse histone H3 as loading control employed polyclonal rabbit anti mouse histone H3 antibodies (1:5000 dilution; ab1971 ABCAM).

### Mass spectrometry

Protein samples were loaded into BoltTM 4–12% Bis-Tris Plus gradient gel (Invitrogen), and the target protein gel band was excised and digested with trypsin as described^40^. After cleanup steps using C18 ZipTips (Millipore), the samples were mixed with an equal amount of matrix solution containing 10 mg/ml R-cyano-4-hydroxycinnamic acid in 0.1% TFA/50% ACN and spotted onto a 384-well stainless steel MALDI target plate (Applied Biosystems, Foster City, CA). An ABI 4800 Proteomics Analyzer MALDI TOF/TOF mass spectrometer (Applied Biosystems) was used to analyze the samples. For protein ID, combined MS and MS/MS data were submitted via GPS Explorer (version 3.6, Applied Biosystems) to Mascot server (version 2.0, Matrix Science). The swissprot database (including 545536 sequences, 194023197 residues) was utilized for the search.

### Immunofluorescence staining

Testes were fixed in 4% paraformaldehyde at 4 degree for 6 hours and dehydrated in 30% sucrose overnight. OCT-embedded testes were cross sectioned at 10 µm thickness and stored in −80 degree. The slides were dried at room temperature overnight, and then washed with PBS three times. After blocking with 10% FBS in PBST, slides were incubated with isolectin IB4-Alexa Fluor488 conjugate (1:200 dilution, Invitrogen) at room temperature for 2 hours in the dark. They were subsequently washed with PBS three times, and sections were mounted and viewed under a fluorescence microscope.

### MRI analysis

Testes of mice with different genotypes from the same litter were fixed with 4% formaldehyde, soaked in PBS overnight before MRI analysis. All MRI experiments were using a 14 Tesla Bruker Ascend 600WB vertical bore magnet, equipped with a MicWB40 micro-imaging probe in combination with a Micro2.5 gradient system. Images were obtained using Paravision 6.0.1 software. Excised testis samples were bedded on top of 1% agarose gel in a 10mm diameter glass tube, and a quadrature coil with an inner diameter of 25mm was used to transmit/receive the MR signals. Fast Low Angle Shot (FLASH) pulse sequence with gradient echo was used to acquire 2D images. Susceptibility weighted images (SWI) were reconstructed to obtain suitable contrast of blood vessels versus seminiferous tubules.

### Sperm collection and protein extraction

Mature male mice (2 to 4 months) were sacrificed, then their cauda epididymides were removed and placed on sterile filter paper to blot away any blood and fluid. The removed cauda epididymides were put in a sperm dish containing 100 μL HTF media, then cut and dissected, and sperms were released. 2 μL of sperms were fixed with 4% formaldehyde, mounted and viewed under a confocal microscope. The sperm proteins were extracted as described^41^ and separated on a 10% SDS-PAGE gel for the Western Blot.

### Data availability

The datasets generated and/or analyzed during the current study are available from the corresponding author on reasonable request.

## Electronic supplementary material

supplementary information

movie 1

movie 2

movie 3
