# Publisher Correction: *Utf1* contributes to intergenerational epigenetic inheritance of pluripotency

**DOI:** 10.1038/s41598-018-20336-x

**Published:** 2018-01-23

**Authors:** Qiuye Bao, Amir Morshedi, Fulu Wang, Sharma Bhargy, Konstantin Pervushin, Wei-Ping Yu, Peter Dröge

**Affiliations:** 1 School of Biological Sciences, Nanyang Technological University, 60 Nanyang Drive, Singapore, 637551 Singapore; 2 Animal Gene Editing Laboratory, Biological Resource Centre, Agency for Science, Technology and Research (A*STAR), Singapore, 138673 Singapore; 3 Nanyang Institute of Structural Biology, Nanyang Technological University, 59 Nanyang Drive, Singapore, 637551 Singapore; 4 Institute of Molecular and Cell Biology, Agency for Science, Technology and Research (A*STAR), Singapore, 138673 Singapore; 5 Present Address: Institute of Diabetes and Regeneration Research, Helmholtz Zentrum München, 85764 Neuherberg, Germany

Correction to: *Scientific Reports* 10.1038/s41598-017-14426-5, published online 03 November 2017

The original HTML version of this Article contained a typographical error in the publication date ‘03 November 2017’ which was incorrectly given as ‘06 November 2017’. This has now been corrected in the HTML version of the Article.

