# Supplementary material for: Utf1 contributes to intergenerational epigenetic inheritance of pluripotency
Source: Sci Rep. 2017 Nov 3;7:14612. doi: 10.1038/s41598-017-14426-5 (PMC5668265; doi:10.1038/s41598-017-14426-5)
Supplement: Supplementary file 1 — supplementary information [file 41598_2017_14426_MOESM1_ESM.pdf]

Utf1 contributes to intergenerational epigenetic inheritance of pluripotency

Qiuye Bao[1], Amir Morshedi[1†], Fulu Wang[2], Sharma Bhargy[1], Konstantin Pervushin[1,3], Wei-Ping Yu[2,4] & Peter Dröge[1,3,*]

[1]School of Biological Sciences, Nanyang Technological University, 60 Nanyang Drive, Singapore 637551, Singapore

[2]Animal Gene Editing Laboratory, Biological Resource Centre, Agency for Science, Technology and Research (A*STAR), Singapore 138673, Singapore

[3]Nanyang Institute of Structural Biology, Nanyang Technological University, 59 Nanyang Drive, Singapore 637551, Singapore

[4]Institute of Molecular and Cell Biology, Agency for Science, Technology and Research (A*STAR), Singapore 138673, Singapore

[†]Present address: Institute of Diabetes and Regeneration Research, Helmholtz Zentrum München, 85764 Neuherberg, Germany

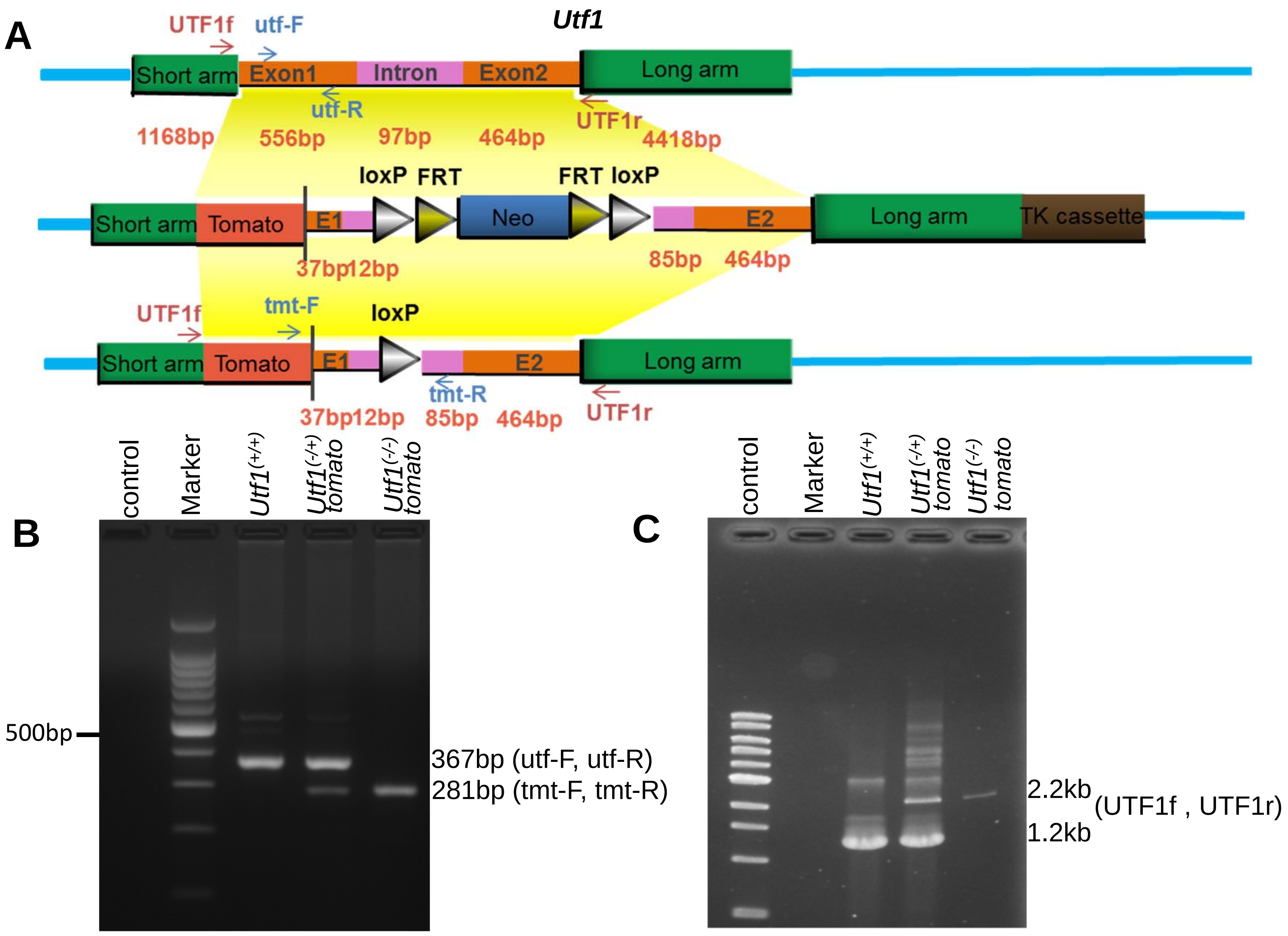

Figure S1 Generation of *Utf1-tomato* reporter mice

(**A**) Design of the target vector for the mouse *Utf1* gene on chromosome 7. Short and long arms of homology to the mouse *Utf1* locus on chromosome 7 are indicated. The final targeted *Utf1-tomato* gene composition in transgenic mice is diagrammed at the bottom. (**B**) Genotyping by PCR. Genomic DNA from *Utf1*$^{(+/+)}$ mice using utf-F and utf-R as primers (A) give raise to a 367bp fragment. Genomic DNA from *Utf1*$^{(-/-)}$*tomato* mice results in a 281bp fragment (using tmt-F and tmt-R as primers); DNA from *Utf1*$^{(-/+)}$*tomato* mice produce both bands. (**C**) PCR product for sequencing the entire 2.2 kb transgene cassette, using UTF1f and UTF1r primers.

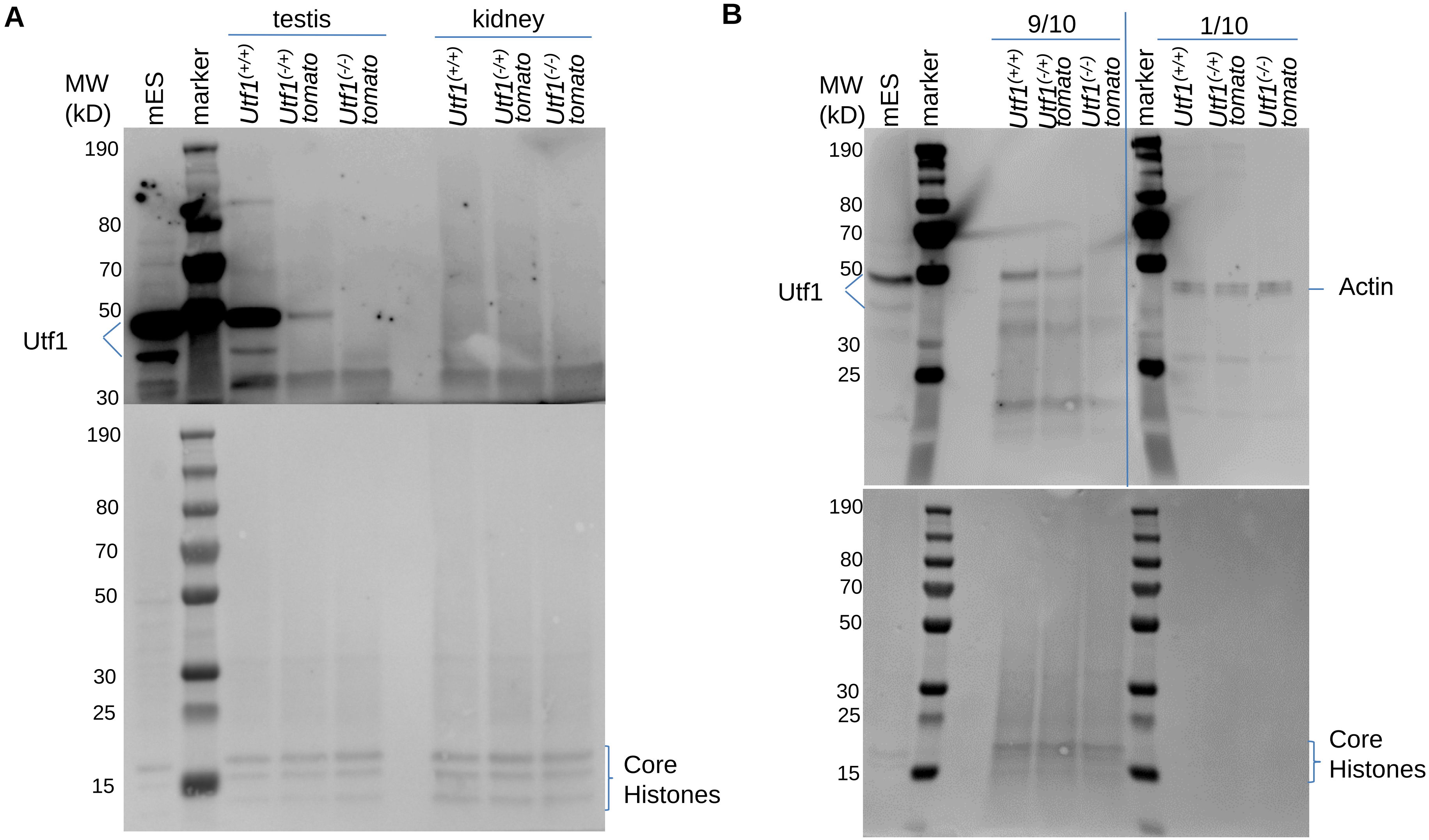

Figure S2. Utf1 expression in embryonic testes of different genotypes by Western Blotting.
Total protein extract from mouse ESCs (mES) was used as positive control (left lane). (**A**) The upper panel shows that Utf1 expression is detectable in *Utf1*$^{(+/+)}$ and *Utf1*$^{(-/+)}$*-tomato* embryonic testes, but not in kidneys because the proein is not expressed in this organ during embryogenesis; the bottom part of the membrane was cut for blotting of another protein. Lower panel: Ponceau S-staining of the uncut PVDF membrane. All embryos analyzed came from the same pregnant female. (**B**) Upper panel: A third set of embryonic testes analyzed for Utf1 expression. 9/10 of the samples were loaded for Utf1 expression, and 1/10 of samples were loaded on the same gel to detect Actin as loading control. *Utf1*$^{(-/+)}$ *tomato* and *Utf1*$^{(-/-)}$*tomato* embryonic testes are from the same pregnant female mouse, while the wild-type lysate came from an age-matched embryo obtained from a different female. Lower panel: Ponceau S-staining as seperate loading control.

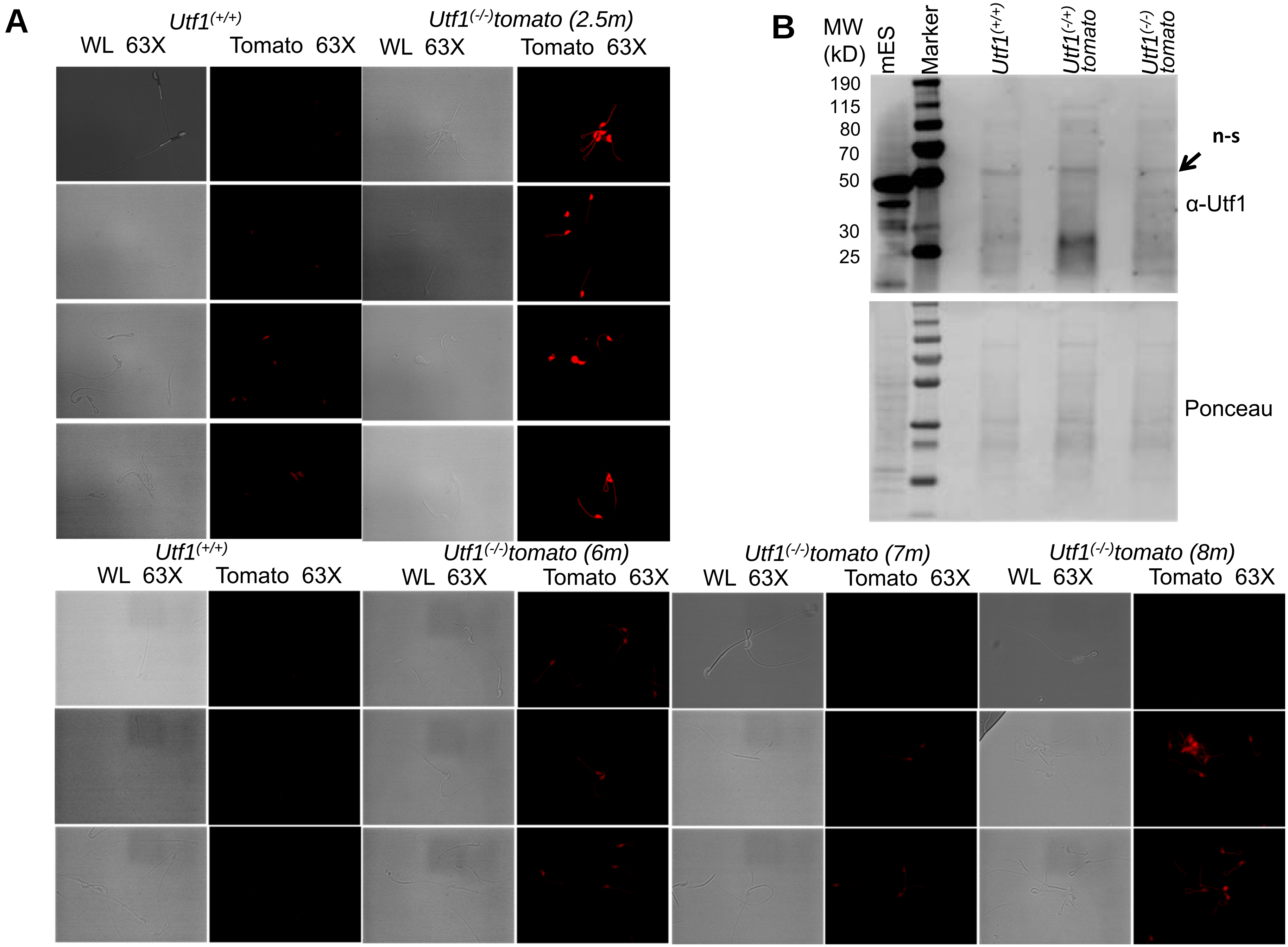

Figure S3. Expression of tomato and Utf1 protein in mature sperm cells.
(**A**) Compared to wild-type control sperm cells (*Utf1*$^{(+/+)}$), those extracted from young *Utf1*$^{(-/-)}$*tomato* males showed strong fluorescent signals, which became more varied in older males (bottom row of panels). (**B**) A seperate set of sperm cells was extracted from mice with the inidcated corresponding *Utf1* genotypes, and lysates were analyzed for Utf1 expression by Western Blotting. n-s.; non-specific WB signal migrating slightly above the Utf1 signal. Lower panel: Ponceau staining of the entire membrane as gel loading control.

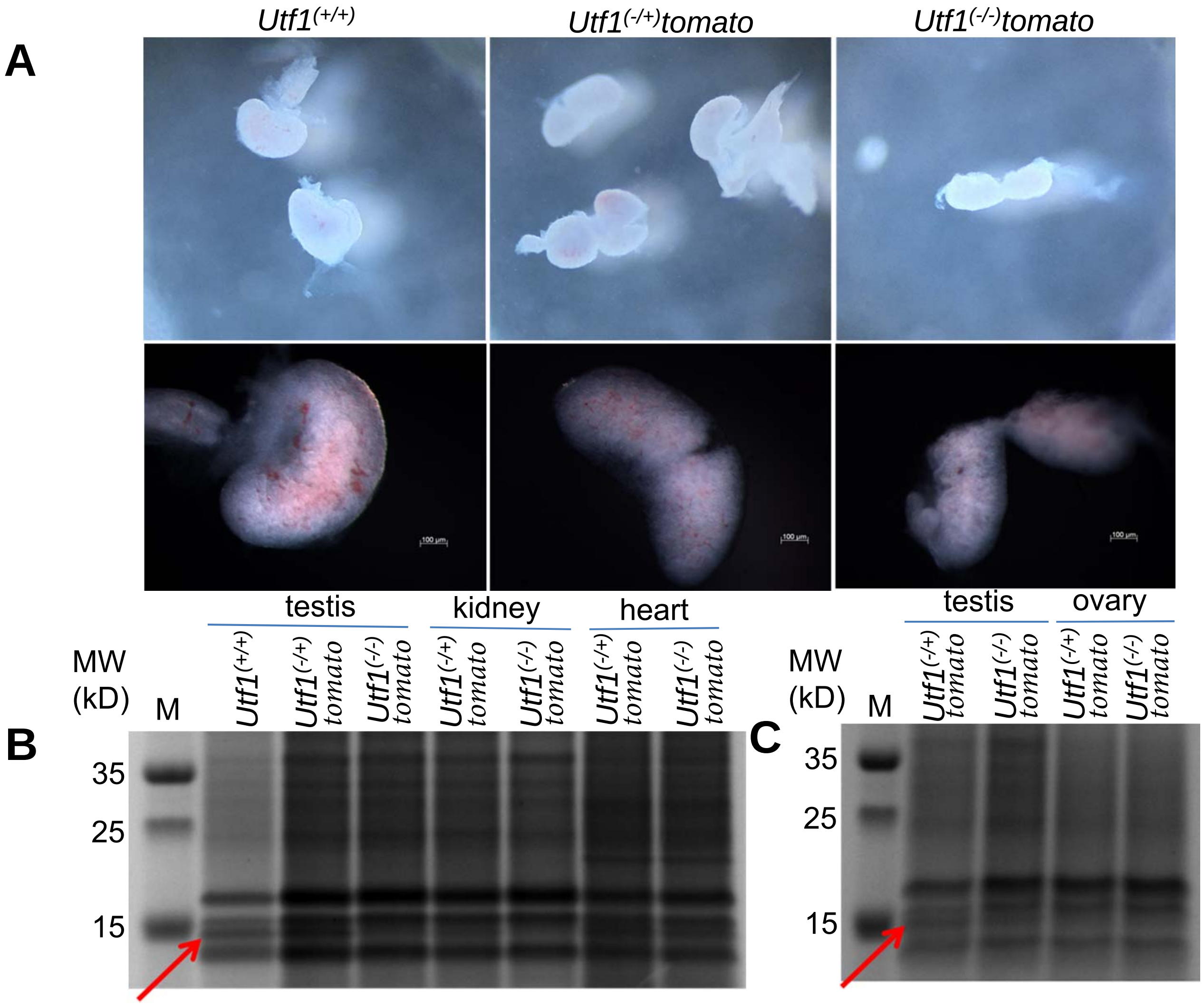

Figure S4 Surface vasculature development of ovaries at 18.5 dpc

(**A**) Surface vasculature of embryonic ovaries with different *Utf1* genotypes. (**B**) SDS-PAGE of total lysates from embryonic testes, kidney and heart showed a prominent 15kD protein (red arrow), which was pronounced in lysates from *Utf1*$^{(+/+)}$ testes and almost undetectable in *Utf1*$^{(-/-)}$*tomato* organs. The protein was identified by MS as hemoglobin α. (**C**) Expression of hemoglobin α (arrow) in testis (*Utf1*$^{(-/+)}$ *tomato*; *Utf1*$^{(-/-)}$ *tomato*) and ovaries (*Utf1*$^{(-/+)}$ *tomato*; *Utf1*$^{(-/-)}$ *tomato*).

Analysis Information

Report Type	Protein-Peptide Summary by Spot	Analysis Type	Combined (MS+MS/MS)
Sample Set Name	160629	Database	Swissprot
Analysis Name	160824_Bao_SPMouse	Creation Date	08/24/2016 12:14:31
Reported By	05/31/2017 14:11:42 - admin	Last Modified	08/24/2016 12:21:49

MS Acq. : Proc. Methods	(Unspecified) : (Unspecified)
Interpretation Method	(Unspecified)

**Gel Idx/Pos**	183/H10	**Instr./Gel Origin**	ak072/160629	**Process Status**	Analysis Succeeded
**Plate [#] Name**	[1] mw1	**Instrument Sample Name**		**Spectra**	11

Rank	Protein Name	Species	Accession No.	Protein MW	Protein Score	Protein Score C. I. %	Total Ion Score	Total Ion C. I. %	Confirmed
1	Hemoglobin subunit alpha OS=Mus musculus GN=Hba PE=1 SV=2		sp|P01942|HBA_MOUSE	15132.8	703	100	624	100	.T.

Peptide Information

Calc. Mass	Obsrv. Mass	± da	± ppm	Start Seq.	End Seq.	Sequence	Ion Score	C. I. %	Modification	Rank	Result Type
1045.5023	1045.4814	-0.0209	-20	33	41	MFASFPTTK			Oxidation (M)[1]		Mascot
1336.6777	1336.7312	0.0535	40	1	12	MVLSGEDKSNIK			Oxidation (M)[1]		Mascot
1336.6777	1336.7312	0.0535	40	1	12	MVLSGEDKSNIK			Oxidation (M)[1]		Mascot
1529.7343	1529.6997	-0.0346	-23	18	32	IGGHGAEYGAEALER	141	100			Mascot
1529.7343	1529.6997	-0.0346	-23	18	32	IGGHGAEYGAEALER					Mascot
1702.9122	1702.8738	-0.0384	-23	2	17	VLSGEDKSNIKAAWGK	99	100			Mascot
1702.9122	1702.8738	-0.0384	-23	2	17	VLSGEDKSNIKAAWGK					Mascot
1819.8762	1819.8354	-0.0408	-22	42	57	TYFPHFDVSHGSAQVK	120	100			Mascot
1819.8762	1819.8354	-0.0408	-22	42	57	TYFPHFDVSHGSAQVK					Mascot
2043.0042	2042.963	-0.0412	-20	13	32	AAWGKIGGHGAEYGAEALER					Mascot
2199.073	2199.0256	-0.0474	-22	42	61	TYFPHFDVSHGSAQVKGHGK	129	100			Mascot
2199.073	2199.0256	-0.0474	-22	42	61	TYFPHFDVSHGSAQVKGHGK					Mascot
2327.168	2327.1194	-0.0486	-21	42	62	TYFPHFDVSHGSAQVKGHGKK	136	100			Mascot

Figure S5: Mass spectrometry identification of the 15kD protein missing in *Utf1*$^{(-/-)}$*-tomato* mouse embryonic testis.
Screen shots of top hits in the MS analysis, which identified the 15 kD protein (see **Figure S4,B**) as hemoglobin α.

Calc. Mass	Obsrv. Mass	± da	± ppm	Start Seq.	End Seq.	Sequence	Ion Score	C. I. %	Modification	Rank	Result Type
2327.168	2327.1194	-0.0486	-21	42	62	TYFPHFDVSHGSAQVKGHGKK					Mascot
2556.2188	2556.1621	-0.0567	-22	18	41	IGGHGAEYGAEALERMFASFPTTK			Oxidation (M)[16]		Mascot

2	Histone H2A type 1-H OS=Mus musculus GN=Hist1h2ah PE=1 SV=3	sp|Q8CGP6|H2A1H_MOUSE	13941.8	67	99.635	43	98.872	.T.

Peptide Information

Calc. Mass	Obsrv. Mass	± da	± ppm	Start Seq.	End Seq.	Sequence	Ion Score	C. I. %	Modification	Rank	Result Type
944.5311	944.5132	-0.0179	-19	22	30	AGLQFPVGR	27	58.571			Mascot
944.5311	944.5132	-0.0179	-19	22	30	AGLQFPVGR					Mascot
1274.6964	1274.6912	-0.0052	-4	19	30	SSRAGLQFPVGR					Mascot
1336.7596	1336.7312	-0.0284	-21	22	33	AGLQFPVGRVHR	16	0			Mascot
1336.7596	1336.7312	-0.0284	-21	22	33	AGLQFPVGRVHR					Mascot
1719.0288	1718.8802	-0.1486	-86	22	36	AGLQFPVGRVHRLLR					Mascot
2059.2637	2058.9573	-0.3064	-149	101	120	VTIAQGGVLPNIQAVLLPKK					Mascot

3	Histone H2A type 1-F OS=Mus musculus GN=Hist1h2af PE=1 SV=3	sp|Q8CGP5|H2A1F_MOUSE	14153	66	99.609	43	98.872	.T.

Peptide Information

Calc. Mass	Obsrv. Mass	± da	± ppm	Start Seq.	End Seq.	Sequence	Ion Score	C. I. %	Modification	Rank	Result Type
944.5311	944.5132	-0.0179	-19	22	30	AGLQFPVGR	27	58.571			Mascot
944.5311	944.5132	-0.0179	-19	22	30	AGLQFPVGR					Mascot
1274.6964	1274.6912	-0.0052	-4	19	30	SSRAGLQFPVGR					Mascot
1336.7596	1336.7312	-0.0284	-21	22	33	AGLQFPVGRVHR	16	0			Mascot
1336.7596	1336.7312	-0.0284	-21	22	33	AGLQFPVGRVHR					Mascot
1719.0288	1718.8802	-0.1486	-86	22	36	AGLQFPVGRVHRLLR					Mascot
2059.2637	2058.9573	-0.3064	-149	101	120	VTIAQGGVLPNIQAVLLPKK					Mascot

4	Histone H2A.J OS=Mus musculus GN=H2afj PE=1 SV=1	sp|Q8R1M2|H2AJ_MOUSE	14037	66	99.581	43	98.872	.T.

Peptide Information

Calc. Mass	Obsrv. Mass	± da	± ppm	Start Seq.	End Seq.	Sequence	Ion Score	C. I. %	Modification	Rank	Result Type
944.5311	944.5132	-0.0179	-19	22	30	AGLQFPVGR	27	58.571			Mascot
944.5311	944.5132	-0.0179	-19	22	30	AGLQFPVGR					Mascot

Figure S5: Mass spectrometry identification of the 15kD protein missing in *Utf1$^{(-/-)}$tomato* mouse embryonic testis.

Screen shots of top hits in the MS analysis, which identified the 15 kD protein (see **Figure S4,B**) as hemoglobin α.

Calc. Mass	Obsrv. Mass	± da	± ppm	Start Seq.	End Seq.	Sequence	Ion Score	C. I. %	Modification	Rank	Result Type
1274.6964	1274.6912	-0.0052	-4	19	30	SSRAGLQFPVGR					Mascot
1336.7596	1336.7312	-0.0284	-21	22	33	AGLQFPVGRVHR	16	0			Mascot
1336.7596	1336.7312	-0.0284	-21	22	33	AGLQFPVGRVHR					Mascot
1719.0288	1718.8802	-0.1486	-86	22	36	AGLQFPVGRVHRLLR					Mascot
2059.2637	2058.9573	-0.3064	-149	101	120	VTIAQGGVLPNIQAVLLPK K					Mascot

5 Histone H2A type 1 OS=Mus musculus GN=Hist1h2ab PE=1 SV=3 — sp|P22752|H2A1_MOUSE 14127 66 99.571 43 98.872 .T.

Protein Group

Histone H2A type 1-K OS=Mus musculus GN=Hist1h2ak PE=1 SV=3 — sp|Q8CGP7|H2A1K_MOUSE 14141

Histone H2A type 3 OS=Mus musculus GN=Hist3h2a PE=1 SV=3 — sp|Q8BFU2|H2A3_MOUSE 14112.9

Peptide Information

Calc. Mass	Obsrv. Mass	± da	± ppm	Start Seq.	End Seq.	Sequence	Ion Score	C. I. %	Modification	Rank	Result Type
944.5311	944.5132	-0.0179	-19	22	30	AGLQFPVGR	27	58.571			Mascot
944.5311	944.5132	-0.0179	-19	22	30	AGLQFPVGR					Mascot
1274.6964	1274.6912	-0.0052	-4	19	30	SSRAGLQFPVGR					Mascot
1336.7596	1336.7312	-0.0284	-21	22	33	AGLQFPVGRVHR	16	0			Mascot
1336.7596	1336.7312	-0.0284	-21	22	33	AGLQFPVGRVHR					Mascot
1719.0288	1718.8802	-0.1486	-86	22	36	AGLQFPVGRVHRLLR					Mascot
2059.2637	2058.9573	-0.3064	-149	101	120	VTIAQGGVLPNIQAVLLPK K					Mascot

6 Histone H2A type 2-C OS=Mus musculus GN=Hist2h2ac PE=1 SV=3 — sp|Q64523|H2A2C_MOUSE 13979.8 64 99.408 43 98.872 .T.

Peptide Information

Calc. Mass	Obsrv. Mass	± da	± ppm	Start Seq.	End Seq.	Sequence	Ion Score	C. I. %	Modification	Rank	Result Type
944.5311	944.5132	-0.0179	-19	22	30	AGLQFPVGR	27	58.571			Mascot
944.5311	944.5132	-0.0179	-19	22	30	AGLQFPVGR					Mascot
1274.6964	1274.6912	-0.0052	-4	19	30	SSRAGLQFPVGR					Mascot
1336.7596	1336.7312	-0.0284	-21	22	33	AGLQFPVGRVHR	16	0			Mascot
1336.7596	1336.7312	-0.0284	-21	22	33	AGLQFPVGRVHR					Mascot
1719.0288	1718.8802	-0.1486	-86	22	36	AGLQFPVGRVHRLLR					Mascot
2059.2637	2058.9573	-0.3064	-149	101	120	VTIAQGGVLPNIQAVLLPK K					Mascot

7 Histone H2A type 2-A OS=Mus musculus GN=Hist2h2aa1 PE=1 SV=3 — sp|Q6GSS7|H2A2A_MOUSE 14086.9 64 99.394 43 98.872 .T.

Figure S5: Mass spectrometry analysis of the 15kD protein that is missing in *Utf1$^{(-/-)}$tomato* mouse embryonic testis. Screen shot of top hits in MS analysis, which identified the 15 kD protein **(Figure S4,B)** as hemoglobin α

Peptide Information

Calc. Mass	Obsrv. Mass	± da	± ppm	Start Seq.	End Seq.	Sequence	Ion Score	C. I. %	Modification	Rank	Result Type
944.5311	944.5132	-0.0179	-19	22	30	AGLQFPVGR	27	58.571			Mascot
944.5311	944.5132	-0.0179	-19	22	30	AGLQFPVGR					Mascot
1274.6964	1274.6912	-0.0052	-4	19	30	SSRAGLQFPVGR					Mascot
1336.7596	1336.7312	-0.0284	-21	22	33	AGLQFPVGRVHR	16	0			Mascot
1336.7596	1336.7312	-0.0284	-21	22	33	AGLQFPVGRVHR					Mascot
1719.0288	1718.8802	-0.1486	-86	22	36	AGLQFPVGRVHRLLR					Mascot
2059.2637	2058.9573	-0.3064	-149	101	120	VTIAQGGVLPNIQAVLLPK K					Mascot

8	Histone H2A type 2-B OS=Mus musculus GN=Hist2h2ab PE=1 SV=3	sp|Q64522|H2A2B _MOUSE	14004.8	56	95.999	43	98.872	.T.

Peptide Information

Calc. Mass	Obsrv. Mass	± da	± ppm	Start Seq.	End Seq.	Sequence	Ion Score	C. I. %	Modification	Rank	Result Type
944.5311	944.5132	-0.0179	-19	22	30	AGLQFPVGR	27	58.571			Mascot
944.5311	944.5132	-0.0179	-19	22	30	AGLQFPVGR					Mascot
1274.6964	1274.6912	-0.0052	-4	19	30	SSRAGLQFPVGR					Mascot
1336.7596	1336.7312	-0.0284	-21	22	33	AGLQFPVGRVHR	16	0			Mascot
1336.7596	1336.7312	-0.0284	-21	22	33	AGLQFPVGRVHR					Mascot
1719.0288	1718.8802	-0.1486	-86	22	36	AGLQFPVGRVHRLLR					Mascot

9	Histone H2AX OS=Mus musculus GN=H2afx PE=1 SV=2	sp|P27661|H2AX_ MOUSE	15133.4	56	95.811	43	98.872	.T.

Peptide Information

Calc. Mass	Obsrv. Mass	± da	± ppm	Start Seq.	End Seq.	Sequence	Ion Score	C. I. %	Modification	Rank	Result Type
944.5311	944.5132	-0.0179	-19	22	30	AGLQFPVGR	27	58.571			Mascot
944.5311	944.5132	-0.0179	-19	22	30	AGLQFPVGR					Mascot
1274.6964	1274.6912	-0.0052	-4	19	30	SSRAGLQFPVGR					Mascot
1336.7596	1336.7312	-0.0284	-21	22	33	AGLQFPVGRVHR	16	0			Mascot
1336.7596	1336.7312	-0.0284	-21	22	33	AGLQFPVGRVHR					Mascot
1719.0288	1718.8802	-0.1486	-86	22	36	AGLQFPVGRVHRLLR					Mascot

10	Histone H2A.Z OS=Mus musculus GN=H2afz PE=1 SV=2	sp|P0C0S6|H2AZ_ MOUSE	13544.5	32	0	27	58.571	.T.

Figure S5: Mass spectrometry analysis of the 15kD protein that is missing in *Utf1*$^{(-/-)}$*tomato* mouse embryonic testis. Screen shot of top hits in MS analysis, which identified the 15 kD protein (**Figure S4,B**) as hemoglobin α.

Protein Group

Histone H2A.V OS=Mus musculus GN=H2afv PE=1 SV=3	sp|Q3THW5|H2AV _MOUSE	13500.5

Peptide Information

Calc. Mass	Obsrv. Mass	± da	± ppm	Start Seq.	End Seq.	Sequence	Ion Score	C. I. %	Modification	Rank	Result Type
944.5311	944.5132	-0.0179	-19	24	32	AGLQFPVGR	27	58.571			Mascot
944.5311	944.5132	-0.0179	-19	24	32	AGLQFPVGR					Mascot

11	Peptidyl-prolyl cis-trans isomerase NIMA-interacting 1 OS=Mus musculus GN=Pin1 PE=1 SV=1	sp|Q9QUR7|PIN1_ MOUSE	18473.1	28	0		.T.

Peptide Information

Calc. Mass	Obsrv. Mass	± da	± ppm	Start Seq.	End Seq.	Sequence	Ion Score	C. I. %	Modification	Rank	Result Type
1519.8003	1519.6721	-0.1282	-84	85	97	EEALELINGYIQK					Mascot
1529.7305	1529.6997	-0.0308	-20	1	13	MADEEKLPPGWEK	2	0			Mascot
1529.7305	1529.6997	-0.0308	-20	1	13	MADEEKLPPGWEK					Mascot
1543.8452	1543.6912	-0.154	-100	71	82	RPSSWRQEKITR					Mascot
1706.9231	1706.8716	-0.0515	-30	57	70	VRCSHLLVKHSQSR			Carbamidomethyl (C)[3]		Mascot
1734.9272	1734.8633	-0.0639	-37	83	97	SKEEALELINGYIQK					Mascot
1740.8374	1740.8374	0	0	130	144	GQMQKPFEDASFALR			Oxidation (M)[3]		Mascot

Figure S5: Mass spectrometry analysis of the 15kD protein that is missing in *Utf1$^{(-/-)}$tomato* mouse embryonic testis. Screen shot of top hits in MS analysis, which identified the 15 kD protein (**Figure S4,B**) as hemoglobin α.

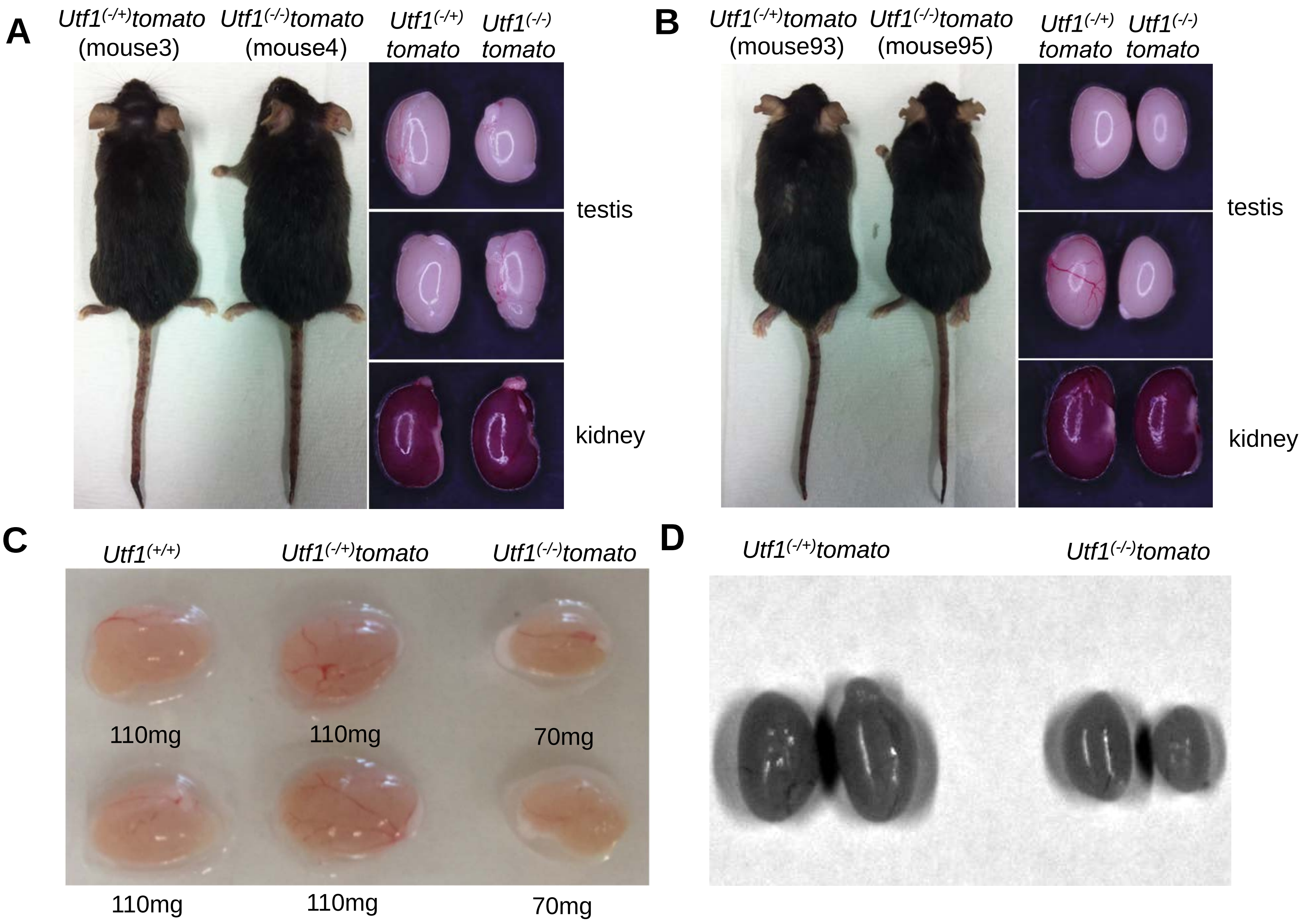

Figure S6. **Testes size in *Utf1*$^{(-/-)}$*tomato* reporter mice**

(**A**) and (**B**) We show two pairs of *Utf1*$^{(-/+)}$*tomato* and *Utf1*$^{(-/-)}$*tomato* mice at the age of 2.5 months and obtained from different litters . The size of these mice and their kidneys was indistinguishable, but the testes size of *Utf1*$^{(-/-)}$*tomato* mice was substantially smaller than those from *Utf1*$^{(-/+)}$*-tomato* mice. (**C**) The weight of individual testis, as indicated, from *Utf1*$^{(-/+)}$ *tomato* and *Utf1*$^{(-/-)}$*tomato* mice (littermates). The age-matched *Utf1*$^{(+/+)}$ mouse was from a different litter. (**D**) A fifth pair of testes of *Utf1*$^{(-/+)}$ *tomato* and *Utf1*$^{(-/-)}$ *tomato* mice obtained from the same litter.

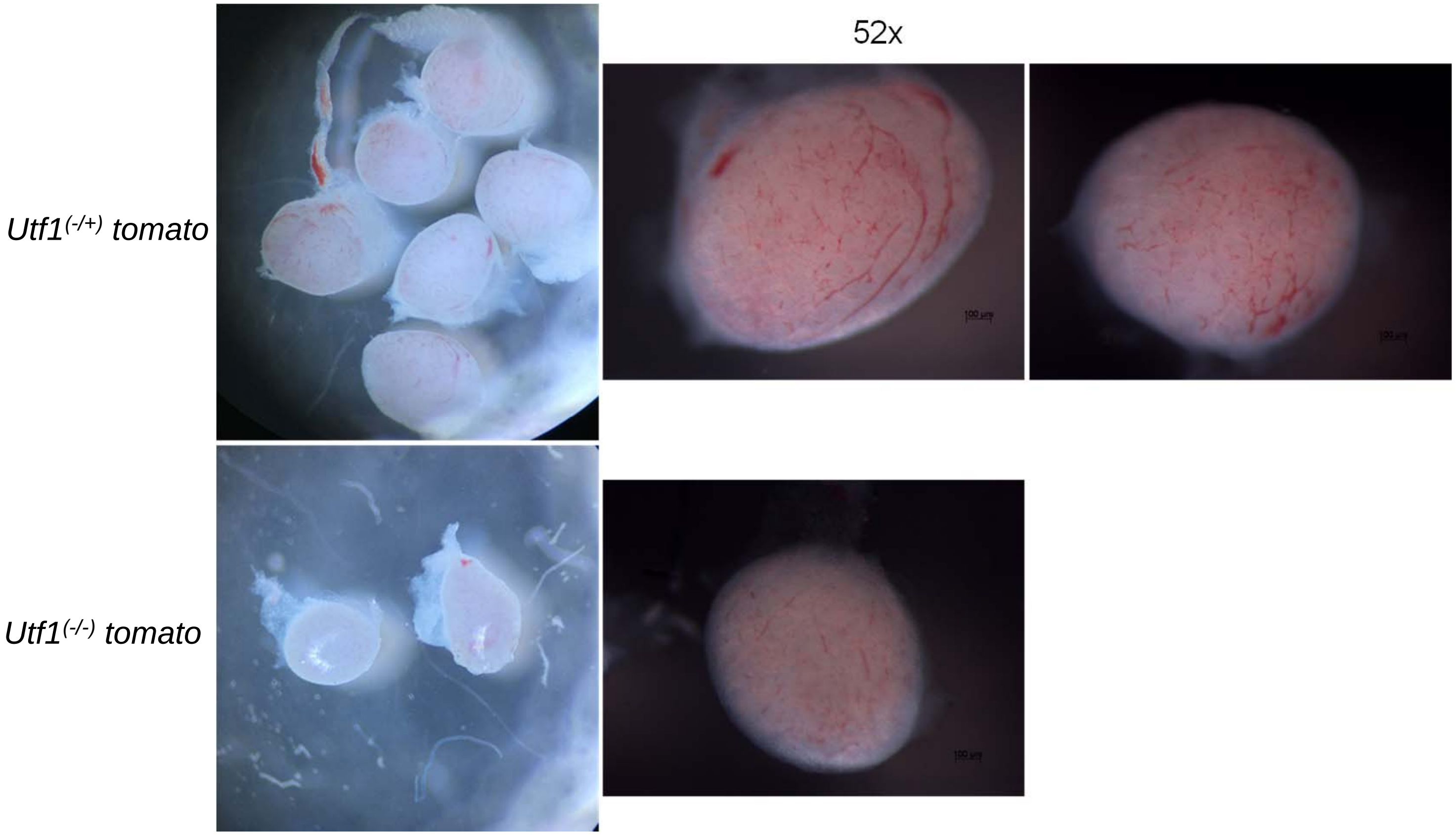

Figure S7. **Testes from *Utf1$^{(-/+)}$-tomato* and *Utf1$^{(-/-)}$-tomato* embryos at 18.5dpc.**

Utf1$^{(-/-)}$-tomato testes were substantially smaller and showed fewer and smaller blood capillaries on the surface, compared to *Utf1$^{(-/+)}$-tomato* testes. Scale bars are shown in the right panels. Also note the reduced visible surface vasculature in *Utf1$^{(-/-)}$-tomato* testes.

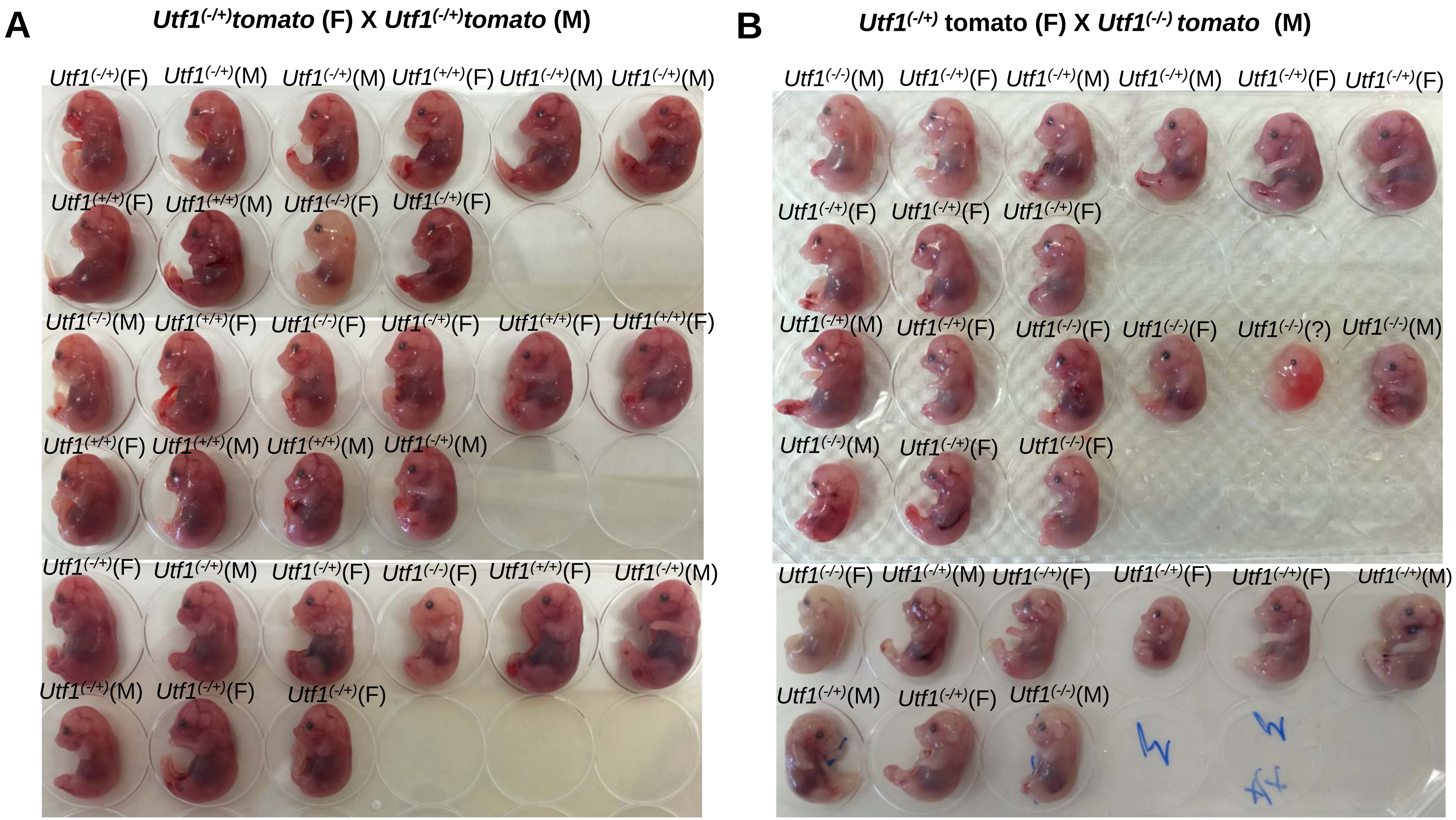

Figure S8. Panel of embryos at 15.5 dpc resulting from *Utf1*$^{(-/+)}$*-tomato* (female, F) X *Utf1*$^{(-/+)}$*-tomato* (male, M) breeding, and from *Utf1*$^{(-/+)}$*-tomato* (F) X *Utf1*$^{(-/-)}$*-tomato* (M) breeding.

(**A**) Embryos from three *Utf1*$^{(-/+)}$*-tomato* females bred with *Utf1*$^{(-/+)}$*-tomato* males; (**B**) Embryos from three *Utf1*$^{(-/+)}$*-tomato* females bred with *Utf1*$^{(-/-)}$*-tomato* males. See text for details.

Cage No.	*Utf1*$^{(-/+)}$ (F) X *Utf1*$^{(-/+)}$ (M)			*Utf1*$^{(-/+)}$ (F) X *Utf1*$^{(-/-)}$ (M)		*Utf1*$^{(-/-)}$ (F) X *Utf1*$^{(-/+)}$ (M)	
	Utf1$^{(+/+)}$	***Utf1***$^{(-/+)}$	***Utf1***$^{(-/-)}$	***Utf1***$^{(-/+)}$	***Utf1***$^{(-/-)}$	***Utf1***$^{(-/+)}$	***Utf1***$^{(-/-)}$
**1**	3	6	0	5	0	6	1
**2**	1	5	0	7	2	7	4
**3**	2	6	1	7	0	9	0
**4**	0	5	0	4	0	12	1
**5**	1	3	0	4	2	3	0
**6**	3	5	1	8	1	5	0
**7**	2	3	0	5	1	1	3
**8**	5	3	0	3	1	5	0
**9**	0	4	0	6	0		
**10**	5	3	0	4	0		
**11**	4	4	0	4	1		
**12**	0	4	0	6	2		
**13**	2	10	3	6	1		
**14**	1	1	0	5	2		
**15**	1	7	0	3	0		
**16**	4	9	0	9	0		
**17**	0	13	0	2	4		
**18**				2	3		
**19**				8	2		
**20**				1	0		
**21**				2	0		
**22**				9	3		
**23**				3	0		
**24**				4	0		
**25**				4	1		
**26**				4	1		
**27**				2	0		
**28**				7	2		
**29**				10	1		
**total**	***34***	***91***	***5***	***144***	***30***	***48***	***9***
**Average genotype No/cage**	***2***	***5.35***	***0.29***	***4.97***	***1.03***	***6***	***1.13***

Table S1: Analysis of postnatal genotypes at 3 weeks

Supplementary Information (Movies)

Movie 1: MRI of adult *Utf1*$^{(+/+)}$ testis

Movie 2: MRI of adult *Utf1*$^{(+/-)}$-*tomato* testis

Movie 3: MRI of adult *Utf1*$^{(-/-)}$-*tomato* testis
